# Exploring microRNA signatures in pediatric non-infectious uveitis: meta-analysis and molecular profiling of patient samples

**DOI:** 10.1007/s13353-024-00922-8

**Published:** 2024-12-19

**Authors:** Olga Wawrzyniak, Dariusz Wawrzyniak, Michał Smuszkiewicz, Paweł Głodowicz, Anna Gotz-Więckowska, Katarzyna Rolle

**Affiliations:** 1 Department of Ophthalmology, Poznan University of Medical Sciences, Augustyna Szamarzewskiego 84, 61-848 Poznan, Poland; 2 Department of Molecular Neurooncology, Institute of Bioorganic Chemistry Polish Academy of Sciences, Zygmunta Noskowskiego 12/14, 61-704 Poznan, Poland

**Keywords:** Pediatric uveitis, MiRNA, Inflammatory eye disease, Non-coding RNAs, Ophthalmology

## Abstract

To find a distinct non-coding RNA characteristic for idiopathic uveitis in the pediatric population. To explore the autoimmune-related miRNA expression profile in pediatric patients with idiopathic uveitis (IU) and juvenile idiopathic arthritis–associated uveitis (JIA-AU) and find a common molecular background for idiopathic uveitis and other autoimmune diseases. The expression levels of miRNAs were analyzed by quantitative real-time PCR using serum samples from patients with idiopathic uveitis (*n* = 8), juvenile idiopathic arthritis–associated uveitis (*n* = 7), and healthy controls. We selected the most promising miRNAs from the original research papers: miR-16-5p, miR-26a-5p, miR-145-5p, and miR-451a as markers for juvenile idiopathic arthritis; miR-23a-3p, miR-29a-3p, miR-140-5p, miR-193a-5p, and miR-491-5p for uveitis in the adult population; and miR-125a-5p, miR-146a-5p, miR-155-5p, miR-223-5p, and miR-223-3p characteristic for both diseases and confirm their expression changes in serum from children with idiopathic uveitis. We comprehensively reviewed the literature enrolling the papers that met the inclusion criteria (miRNA and non-infectious uveitis/juvenile idiopathic arthritis) and performed target prediction analysis of appoint miRNAs. It additionally confirmed that altered miRNAs target the immunologically involved genes. Immunological-involved miRNAs such as miR-146a-5p and miR-155-5p show diverse expression levels in different patients as they interact with multiple targets. miR-204-5p is downregulated in both patient groups compared to healthy controls. miR-204-5p and miR-155-5p are candidates for molecular markers of autoimmune uveitis. We did not identify the miRNAs specific only to idiopathic uveitis, but for the first time in the pediatric population, we confirmed that this disease entity shares a molecular basis with other autoimmune diseases. Further studies are required to elucidate the molecular interactions among miRNAs, cytokines, and transcription factors within the intricate immune response, particularly in the eye.

**Supplementary Information:**

The online version contains supplementary material available at 10.1007/s13353-024-00922-8.

## Introduction

Uveitis is the inflammation of the uveal membrane of the eye. It can be caused by infectious pathogens or be autoimmune or idiopathic. Pediatric patients represent 5–16% of all patients with uveitis (Holland and Stiehm 2003; Kump et al. 2005). The incidence and prevalence differ between countries and populations, and there are about 4–5 new cases per year per 100,000 children in developed countries (Paivonsalo-Hietanen et al. 2000; Edelsten et al. 2003). Although it can be categorized as a rare disease, it remains an important cause of irreversible blindness (Hoogewoud and C.J., Rossi D., Koryllou A., Guex-Crosier C., Ezziat S., Hofer M., Guex-Crosier Y. 2021; Richter et al. 2015). According to the Standardization of Uveitis Nomenclature (SUN) criteria, uveitis description includes onset, duration, course of inflammation, and anatomical localization (Jabs et al. 2005). Ninety-five percent of uveitis in children is non-infectious with idiopathic uveitis (IU) and juvenile idiopathic arthritis–associated uveitis (JIA-AU) as the most common (Kump et al. 2005; Nagpal et al. 2008). It is usually insidious, chronic, and persistent in duration (Maleki et al. 2022).

Due to often delayed diagnosis, difficult examination, and limited treatment options in young patients, pediatric uveitis can lead to severe chronic inflammation, a higher risk of developing ocular complications, and permanent vision loss (Smith et al. 2009; Cunningham et al. 2016). Major undesirable outcomes of the disease and its treatment are posterior synechiae, secondary cataract, secondary glaucoma, hypotony, and maculopathy. All can cause amblyopia (Gautam Seth et al. 2021). Furthermore, the long-term effects of this condition and the use of topical and systemic medications over a long period impact vision-related quality of life and function (Angeles-Han 2015). Despite the improving guidelines for the management of patients with autoimmune diseases, pediatric uveitis remains a vision-threatening condition (Gautam Seth et al. 2021).

Juvenile idiopathic arthritis is the most frequent rheumatic disease in childhood, with an annual incidence between 8 and 22.6 per 100,000 children and prevalence rates between 70 and 401 per 100,000 children (Gowdie and Tse 2012). The diagnosis merges the group of conditions with a common feature: arthritis. The International League of Associations for Rheumatology (ILAR) criteria for the classification of JIA distinguishes seven subtypes of this disease. Oligoarticular arthritis and polyarticular arthritis constitute about 45% of all cases and are strongly associated with asymptomatic uveitis in nearly 45% of patients, especially with the presence of antinuclear antibodies (ANA) (Petty, et al. 2001). Acute symptomatic uveitis usually correlates with enthesitis-related arthritis and HLA-B27 occurrence (Zaripova et al. 2021). Different subtypes of JIA are often considered to be the childhood onset of rheumatological diseases in adults. Polyarticular RF + JIA usually develops into rheumatoid arthritis (RA), and enthesitis-related arthritis with positive HLA-B27 status passes into ankylosing spondylitis (AS) (Zaripova et al. 2021).

Pediatric patients diagnosed with juvenile idiopathic arthritis are under ophthalmological supervision, so the early signs of uveitis can be spotted and properly treated. Also, the general treatment of rheumatoid disease helps with maintaining ocular inflammation. Although several guidelines define the current and most efficient course of follow-up in JIA-AU patients, a group of children with idiopathic uveitis or with uveal inflammation before the rheumatoid diagnosis remains challenging for clinicians (Angeles-Han et al. 2019; Martini et al. 2019). As we meet these patients in our daily practice, we decided to focus on the pediatric population in our research. The lack of a rheumatoid diagnosis makes it difficult to introduce biological treatment because the refund of therapy with biological drugs was dependent on the co-occurrence of arthritis. Before inclusion, the side effects of immunosuppressive or immunomodulating drugs should also be taken into account. In adults, the cooperation of the patient with the doctor, the expected length of future therapy, the impact of general drugs on the adult organism, and the fact that, for example, adalimumab has been registered for the treatment of uveitis without another diagnosis of an autoimmune disease, make the management of adult patients less difficult than children. Finding a molecular marker that will accelerate the diagnosis of a generalized autoimmune process will allow faster implementation of effective and targeted treatment. As the childhood diagnosis of autoimmune disease often becomes the “adult equivalent,” the findings in our research can be accelerated to the adult population as well.

In the adult population, Rosenbaum et al. showed in gene expression studies that patients diagnosed with idiopathic uveitis share a common transcript signature with patients diagnosed with autoimmune disease. To our knowledge, there are only four studies that have examined the miRNA profile in types of uveitis other than Behçet’s disease in the adult population and one study of the miR146a SNP in pediatric uveitis. Most studies on non-coding RNAs explore Behçet’s disease (BD) and Vogt-Koyanagi-Harada disease (VKH). These are conditions that we rarely see in our population, especially in children. There are no studies that would compare directly the miRNA profile in idiopathic uveitis with another autoimmune disease, but the findings of Rosenbaum et al. and Li et al. on expression patterns in uveitic patients allow us to assume the similar differences and similarities at the non-coding RNA level (Rosenbaum et al. 2021a; Li, et al. 2008).

Non-coding RNAs are a heterogeneous group of small RNA particles: microRNAs (miRNAs), long-non-coding RNAs, and circular RNAs. All regulate gene expression at the posttranscriptional level controlling different cellular mechanisms (Saliminejad et al. 2019). Many miRNAs identified in humans are conserved in other animals. These short, ~ 22 nt long particles interact with mRNA and influence developmental processes and diseases (Bartel 2018). Loss-of-function studies disrupting miRNA genes in mice and rats have revealed their involvement in immunological processes, such as the increased proliferation of progenitors and hyperactive neutrophils and macrophages, inflammatory cytokine production, or Th17 cell activation (Qayum et al. 2016; Rodriguez et al. 2007). Changes of the miR-93, miR-155, miR-146a, miR-182, and miR-223 expression levels modify numerous protein transcription, for example, signal transducer and activator of transcription 3 (STAT3), interleukin-1 receptor-associated kinase 4 (IRAK4), or Forkhead box O3 (FOXO3) involved in immune reaction in induced non-infectious uveitis (Zhang et al. 2020; Wei et al. 2019; Muhammad et al. 2019; Ishida et al. 2011; Hsu et al. 2017; Escobar et al. 2013). In cardiovascular and neurological pathologies and oncological processes, the participation of miRNA is well explored (Fatimy et al. 2021; Hata and Kashima 2016; Lu et al. 2008). In autoimmune diseases, they are increasingly attracting attention as potential markers of disease activity and as targets for therapy (Singh et al. 2013). In addition to miRNA, lncRNA and circular RNA also play a role in immunological pathways by interacting with mRNA, acting as decoy particles, or producing proteins with a similar or different activity to their main protein (Mattick et al. 2023; Liu et al. 2019; Wawrzyniak et al. 2018).

Detailed information about non-coding RNA genesis and function in ocular disorders authors presented in previous publications (Wawrzyniak et al. 2018, 2020).

In the present study, we determined an expression profile pattern of inflammatory microRNAs in the blood of pediatric non-infectious uveitis patients and compared it with previous studies performed on samples from adult patients. We also summarized for the first time the molecular background and revealed the similarities and differences in miRNA expression levels between subgroups of non-infectious uveitis in children. We highlighted a set of microRNAs that may have a regulatory function in pediatric uveitis. Thus, we have identified a mechanism caused by altered expression of miRNAs that may be related to these diseases, providing new insights into potential diagnostic and therapeutic targets.

## Materials and methods

### Patients

This study was approved by the Bioethics Committee at the Poznań University of Medical Sciences (NR 951/16), and informed consent was obtained from all parents/legal guardians of the patients and patients above 16 years old.

The study included 15 patients with non-infectious uveitis and 3 healthy controls between 6 and 18 years old, admitted to the Pediatric Ophthalmology Clinic of Poznań University of Medical Sciences between 2019 and 2022.

The epidemiological data of the patients are summarized in Table 1. Additional information on the individual patients is summarized in Supplementary Table S1.

**Table 1:** Clinical characteristics of the cohorts investigated in this study

	**JIA-AU**	**IU**	**HC**
Number of patients	7	8	3
Age at the diagnosis, mean ± SD	6.4 ± 4	12.8 ± 2.9	N/A
Age at sample collection, mean ± SD	14 ± 3.2	15 ± 2	8.7 ± 1.5
Male/female	3/4	5/3	1/2
ANA + (Number of tested patients)	7 (7)	1 (5)	N/A
HLA-B27 + (Number of tested patients)	1 (2)	0 (5)	N/A

JIA-AU juvenile idiopathic arthritis–associated uveitis, IU idiopathic uveitis, HC healthy control, N/A not applicable

### Extraction of total RNA and DNase treatment

The patient’s blood samples were diluted at a 1:1 ratio in phosphate-buffered saline (PBS). Subsequently, 750 µl of TRI Reagent® solution was added to 250 µl of the blood dilution, followed by a 5-min incubation period at room temperature. The lysate was gently mixed to ensure thorough homogenization. Two hundred microliters of chloroform was added to the TRIzol-blood mixture, followed by an incubation period of 10 min with repeated mixing to facilitate phase separation. The aqueous and organic particles were separated by centrifugation for 15 min at 13,000 rpm at 4 °C. The supernatant containing the RNA was carefully transferred to a new Eppendorf tube, and isopropanol was added to the supernatant at an equivalent volume to precipitate the RNA. The samples were incubated overnight at − 20 °C or for 3 h at − 80 °C and centrifuged at 13,000 rpm for 30 min at 4 °C to pellet the RNA. The RNA pellet was washed with 1 ml of cold 80% ethanol and centrifuged at 13,000 rpm 4 °C for 10 min. After removal of the ethanol, the RNA pellet was air-dried for 10 min at room temperature and resuspended in 20 µl of double-distilled, sterile water. The quality of extracted RNA was measured by NanoDrop 2000 spectrophotometer and confirmed by the agarose gel electrophoresis. Afterward, RNA samples were subjected to DNase I treatment using a ready-to-use DNA-free™ Kit DNAse Treatment & Removal (Thermo Fisher Scientific).

### Polyadenylation reaction, miRNA reverse transcription, and quantitative RT-qPCR

The reverse transcription for miRNA was performed in two steps. First, 500 ng of total RNA was polyadenylated by using *E.coli* poly(A) polymerase, followed by reverse transcription for miRNA using AffinityScript One-Step RT-PCR Kit (Agilent Technologies) with an anchored oligo(dT) primer, both according to the manufacturer’s protocol. Complementary DNA (cDNA) was used as a template in real-time quantitative reverse transcription PCR (qRT-PCR), with the use of CFX96 Real-Time System thermal cycler (Bio-Rad), in three technical replicates. Relative expression was analyzed in the CFX96 Real-Time System thermal cycler (Bio-Rad) software version 2.0. In the case of miRNA normalization, the mean level of ACTN1, GAPDH, HPRT, and SDHA as an endogenous control was used. A list of sequences of primers 5′−3′ is presented in Supplementary Table S2. Statistical significance was calculated using an unpaired *t*-test with Welch’s correction, *p ≤ 0.05, **p ≤ 0.01, ***p ≤ 0.001, ****p ≤ 0.0001. Bars representing not significant results (*p* > 0.05) are not shown on the graphs.

### Selection of candidate microRNAs

The 19 microRNAs tested by qRT-PCR in blood samples were selected from a literature review. This literature research was performed in 2019 on the PubMed database and focused on determining a list of microRNAs with potential or known functions in idiopathic uveitis disease, as well as in autoimmune diseases. This biased approach to select candidate microRNAs was intentionally used to highlight potential microRNAs involved in inflammation, the main biological feature of uveitis.

### *Meta*-analysis

To better organize our work, we follow the Preferred Reporting Items for Systematic reviews and Meta-Analyses (PRISMA) 2021 guidelines (Page et al. 2021). Literature searches were conducted using the PubMed database. Considering there was only one publication on non-coding RNAs and pediatric uveitis, we expanded the search strategy. The combinations of search terms included “micro/mi RNA,” “non-coding RNA,” “pediatric uveitis,” “uveitis in children,” “uveitis,” “autoimmune uveitis,” “noninfectious uveitis,” and as pediatric uveitis mostly coexists with juvenile idiopathic arthritis also with “juvenile idiopathic arthritis.”

Original research papers on miRNAs in non-infectious uveitis and juvenile idiopathic arthritis that met the inclusion criteria were thoroughly examined. The inclusion and exclusion criteria are summarized in Table 2.

**Table 2:** Inclusion and exclusion criteria

Inclusion criteria	Exclusion criteria
Original paper	Review
Human samples	Animal models
Idiopathic uveitis, autoimmune uveitis, juvenile idiopathic arthritis	Infectious uveitis
miRNA expression level	SNPs

* SNPs* single nucleotide polymorphisms

### Prediction of miRNA’s targets

Target annotation analysis and network visualization were performed using the R environment (version 4.4, https://www.r-project.org) and appropriate packages according to corresponding reference manuals. Identification of miRNA/gene regulatory interactions was performed in silico between selected miRNAs and uveitis and juvenile arthritis–associated genes harvested from DisGeNET 7.0 database (https://www.disgenet.org/) (Pinero et al. 2020, 2017, 2015), using multiMiR 1.26.0 package (https://bioconductor.org/packages/release/bioc/html/multiMiR.html) (Ru et al. 2014). The analysis included the identification of both experimentally validated (miRecords, miRTarBase, and TarBase databases) and predicted (DIANA-microT-CDS, ElMMo, MicroCosm, miRanda, miRDB, PicTar, PITA, and TargetScan databases) miRNA/gene interactions. The regulatory network visualization with obtained interactions was performed using Cytoscape v3.10.2 software (https://cytoscape.org/) and miRTargetLink 2.0 (Shannon et al. 2003; Kern et al. 2021).

### Enrichment analysis

Pathway enrichment analysis was performed using the ToppGene Suite (https://toppgene.cchmc.org/enrichment.jsp), where a list of selected miRNAs and a list of genes with which they interact were introduced (Chen et al. 2009). The default *Homo sapiens* genome was used as a background. Terms of the Kyoto Encyclopedia of Genes and Genomes (KEGG) pathways (Kanehisa and Goto 2000), REACTOME (Milacic et al. 2024), and Gene Ontology (GO) categories (Ashburner et al. 2000) were searched.

### Statistical analysis

Results at *p* < 0.05 were considered statistically significant. Statistical analyses were performed in GraphPad Prism 8.0.1 (GraphPad Software, San Diego, CA, USA). The analyses included the Shapiro–Wilk test (to assess the compliance of the examined variables with the normal distribution) and the Student’s *t*-test for variables on a quantitative scale (data presented as an average). GraphPad Outlier calculator (https://www.graphpad.com/quickcalcs/grubbs1/) was used to determine the significance of the most extreme values. Significant outliers, *p* < 0.05, were not shown on the graphs (Supplementary Table 3).

## Results

### miRNA expression profile in blood samples

**Fig. 1:**
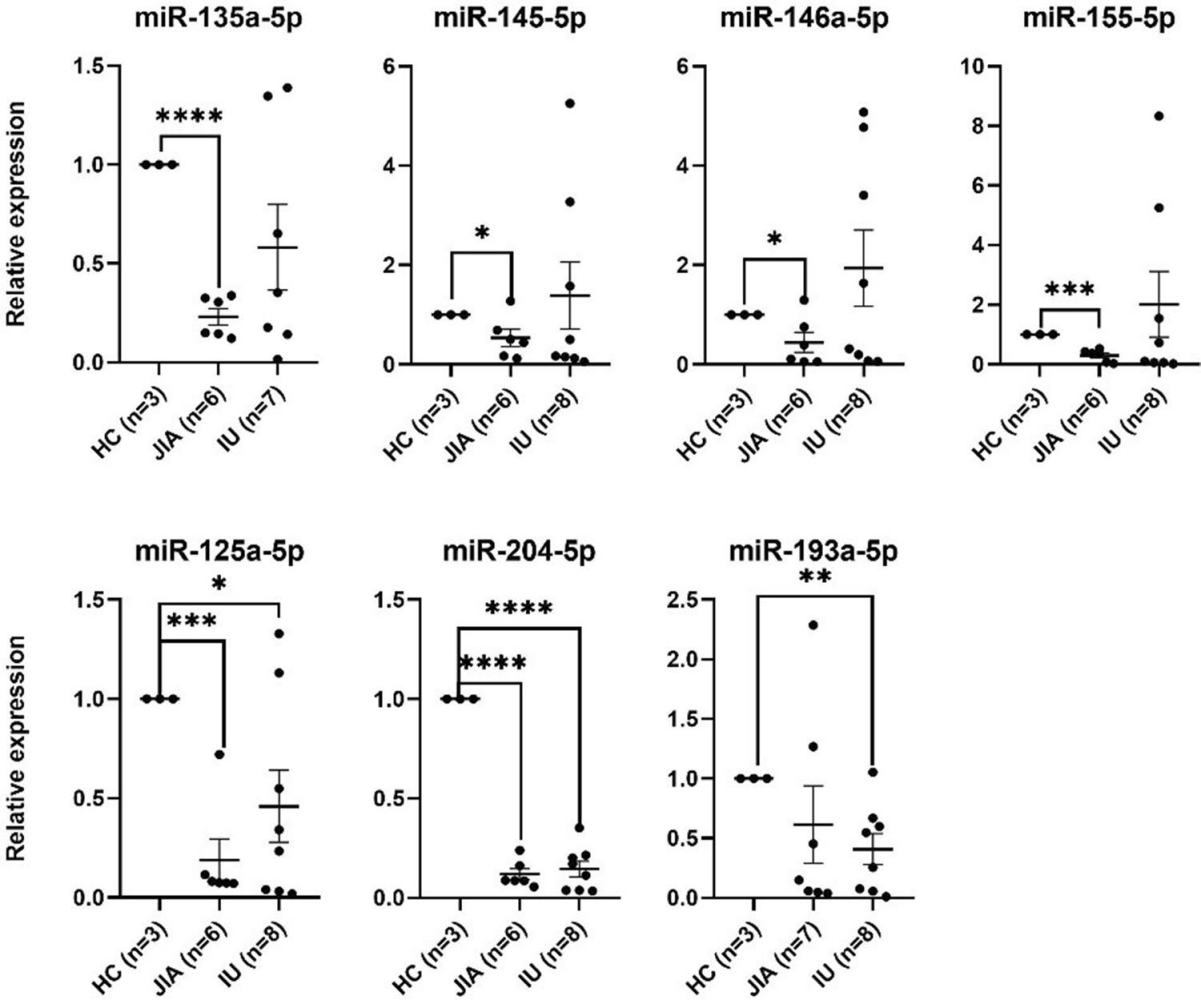
Differentially expressed miRNAs using qRT-PCR between JIA-AU and IU patients and healthy donors. *n* represents number of patients without significant outliers. Statistics was performed with unpaired *t*-test with Welch’s correction, *p ≤ 0.05, **p ≤ 0.01, ***p ≤ 0.001, ****p ≤ 0.0001. Bars representing not significant results (*p* > 0.05) are not shown on the graphs

**Fig. 2:**
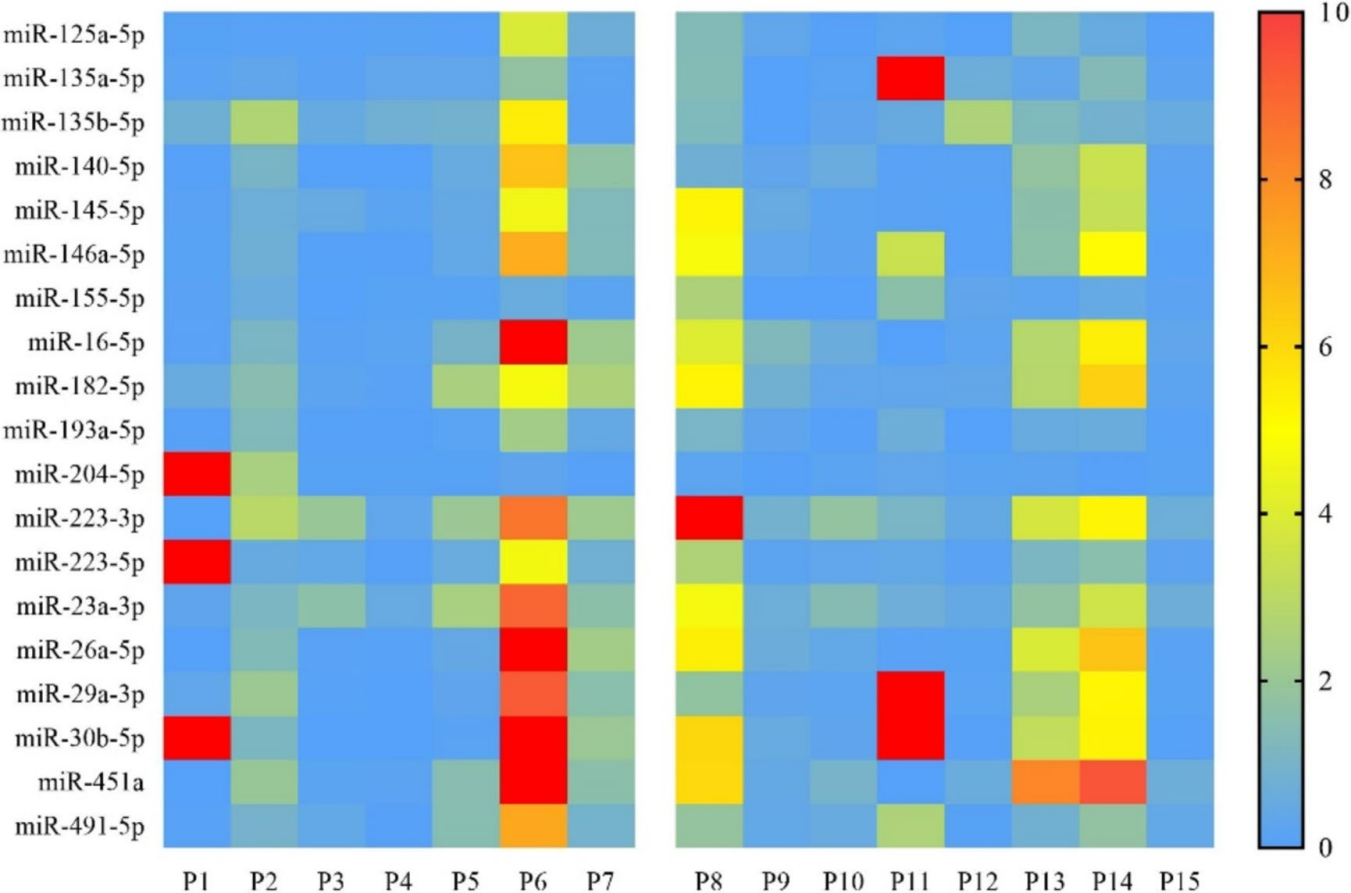
Heatmap showing differential miRNA expression in diseased *vs* normal control group. The left panel represents JIA-AU patients, and the right panel represents IU patients. The green color indicates lower than mean intensity, and red represents higher than mean intensity. Each row represents a miRNA, and each column represents a patient’s sample (*p* < 0.05, logFC >|1|)

To establish the molecular profile of miRNAs, we analyzed the expression of 19 preselected microRNAs in the 18 blood samples from uveitis (15) and healthy (3) patients by qRT-PCR (Figs. 1 and 2). The expression level of selected miRNAs in juvenile idiopathic arthritis–associated uveitis (JIA-AU) patients is not identical in all subjects (Fig. 1). This may be because this group includes patients with different subtypes of JIA who are treated with immunosuppressive and immunomodulatory drugs in various combinations. For patients with JIA-AU (5 upregulated and 14 downregulated) and for patients with IU (13 upregulated and 6 downregulated), miRNAs were examined compared with healthy controls. Among these miRNAs, four upregulated miRNAs (miR-182-5p, miR-223-3p, miR-23a-3p, and miR-30b-5p) and five downregulated miRNAs (miR-125a-5p, miR-135a-5p, miR-135b-5p, miR-193a-5p, and miR-204-5p) were common to the JIA and IU. The rest of the studied miRNAs, such as miR-140-5p, miR-145-5p, miR-146-5p, miR-155-5p, miR-16-5p, miR-26a-5p, miR-29a-3p, miR-451a, miR-491-5p, and miR-223-5p, show an opposite pattern in JIA-AU and IU (Fig. 2).

### *Meta*-analysis

**Fig. 3:**
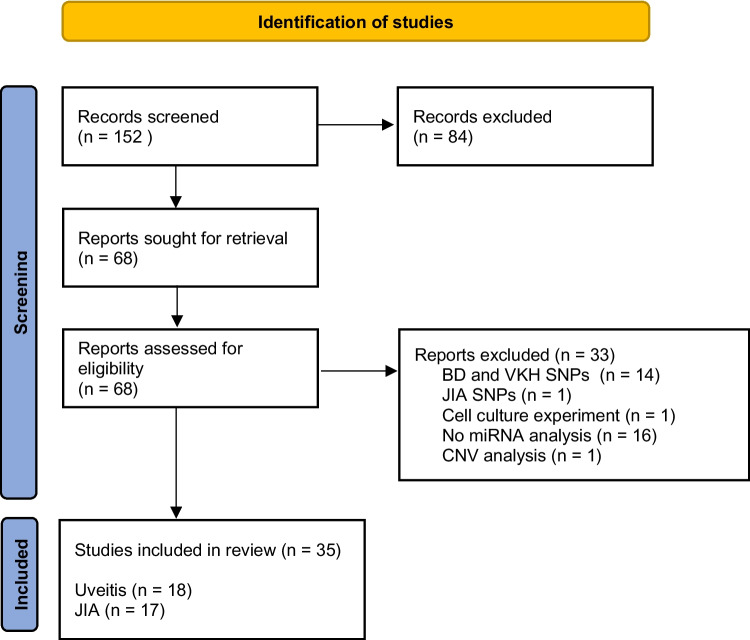
Identification of the studies included in the meta-analysis (search process based on PRISMA 2021). BD, Behçet’s disease; VKH, Vogt-Koyanagi-Harada disease; JIA, juvenile idiopathic arthritis; SNPs, single nucleotide polymorphisms; CNV, copy number variation

One of the aims of the present study was to perform a meta-analysis of miRNA expression profiling studies in human uveitis samples to complement and compare data from RT-qPCR analysis performed on samples from children suffering from uveitis. One hundred fifty-two papers were obtained using combinations of the entries described above. Of these, 68 met the inclusion criteria listed in Table 2. Thirty-five original research papers were included in the comprehensive review, 17 on juvenile idiopathic arthritis, and 18 on non-infectious uveitis. Our search included English-language publications from 2000 to November 2023. Figure 3 based on PRISMA 2021 guidelines shows the literature selection process.

#### miRNA in non-infectious uveitis

The potential use of microRNAs as markers and predictors of specific types of uveitis has been best explored in Behçet’s disease (BD) and Vogt-Koyanagi-Harada disease (VKH) (Yu et al. 2014; Hou et al. 2016; Na et al. 2016; Puccetti et al. 2018; Qi et al. 2013). These two subtypes of uveitis are rare in children. Behçet’s disease in pediatric patients mostly starts in late childhood, and a full diagnosis with all criteria met is usually possible after age 16 (Citirik et al. 2009; Andreoli and Foster 2006). It is common in Turkey, Japan, China, Saudi Arabia, Israel, and Iraq but rare in most developed countries except for the Mediterranean (Tsirouki et al. 2018). The well-explored fields in BD and VKH are miR-146a SNPs as well as miR-196a2 and miR-182 (Yu et al. 2014; Qi et al. 2013; Zhou et al. 2012a, 2012b; Ibrahim et al. 2019; Abdelaleem et al. 2021; Kamal et al. 2022; Oner et al. 2015). As we focused on pediatric patients and miRNA expression levels, we excluded research papers on SNP variants in BD and VKH.

The only study that includes pediatric patients with uveitis and miRNAs explores the miR-146a SNPs in the Han Chinese population. In the group of 520 patients and 1204 healthy controls included in the study, the researchers found that the frequencies of the rs2910164 CC genotype and the C allele of miR-146a were significantly decreased in patients compared to healthy controls. This suggests that miR-146a may be a susceptibility factor for pediatric uveitis. They also correlated the SNP rs10893872 of Ets-1, an oncogene and transcription factor, with uveitis predisposition. The CC genotype and C allele frequencies of this SNP were significantly increased in patients compared to controls. Ets1 can bind to the miR-146a promoter region and significantly affect miR-146a activity (Wei et al. 2014).

To extract the most promising non-coding RNAs involved in non-infectious ocular inflammation, we had to extend our research to adult patients in this field. Among 19 studies performed on human samples, only 5 were conducted on serum, 1 on vitreous, and the rest on peripheral blood mononuclear cells (PBMCs). Most papers explore miRNA expression in Behçet’s disease or in combination with Vogt-Harada-Koyanagi disease. Only four studies investigate other types of uveitis: HLA-B27-associated acute anterior uveitis (AAU), idiopathic intermediate uveitis (IIU), anterior uveitis associated with ankylosing spondylitis, or non-infectious posterior and panuveitis (Asakage et al. 2020; Saito et al. 2022; O’Rourke et al. 2019; Verhagen et al. 2018). Verhagen and colleagues discovered and independently confirmed a group of microRNAs associated with non-infectious uveitis (NIU). miR-140-5p, miR-491-5p, miR-223-3p, miR-223-5p, miR-193a-5p, miR-29a-3p, and U6 snRNA showed strong correlation, prompting researchers to treat them as single miRNA cluster associated with uveitis. Six of these miRNAs were found to target a total of 37 genes. Analysis of these common gene targets revealed significant involvement in pathways related to inflammation and ocular biology (Verhagen et al. 2018).

Research on miR-146a expression in peripheral blood mononuclear cells (PBMCs) from Behçet’s disease (BD) and acute anterior uveitis (AAU) patients has yielded mixed results. Three studies in BD patients and one in AAU patients found reduced miR-146a expression, consistent with its potential anti-inflammatory role (Puccetti et al. 2018; Ahmadi et al. 2019; Kolahi et al. 2018; Yang et al. 2017). However, O’Rourke and colleagues observed increased levels of miR-146a, suggesting that it acts as a regulator of the immune response, in contrast to the pro-inflammatory activity of miR-155. These findings suggest a possible feedback mechanism between the pro-inflammatory miR-155 and the regulatory miR-146a, mediated by Toll-like receptor (TLR) signaling (O’Rourke et al. 2019). Similarly, miR-155 expression is usually upregulated in most studies conducted in PBMCs from BD and AAU patients (Na et al. 2016; O’Rourke et al. 2019; Kolahi et al. 2018). Yet, Zhou et al. showed that miR-155 is downregulated in PBMCs and CD4 + cells in the active phase of BD (Zhou et al. 2012b). In addition, Ahmadi et al. reported that both miR-155 and miR-146a expression were decreased in BD patients, which may play a role in the reduced number of Treg cells noted in BD patients (Ahmadi et al. 2019). This evidence supports the idea that miRNA expression may fluctuate during different stages of the disease and possibly in response to treatment.

MiRNAs have also been investigated for their diagnostic potential in intraocular lymphoma and uveitis (Tuo et al. 2014). Expression profiles of miR-155, miR-200, and miR-22 were found to distinguish between the two diseases. Table 3 summarizes papers about non-infectious uveitis enrolled in this study.

**Table 3:** miRNA in non-infectious uveitis. *BD* Behçet’s disease, *VKH* Vogt-Kyanagi-Harada disease, *HC* healthy control, *AAU* acute autoimmune uveitis, *IIU* idiopathic intermediate uveitis, *BU* birdshot uveitis, *SS* suspected sarcoidosis, *PPU* panuveitis/posterior uveitis, *S* sarcoidosis, *AIU* anterior idiopathic uveitis, *AAU AS* ± acute anterior uveitis with or without ankylosing spondylitis, *PBL* primary B-cell lymphoma, *PBMC* peripheral blood mononuclear cells

Patients and diagnosis	miRNA upregulated	miRNA downregulated	Sample	miRNA identification method	Reference
Patients *n* = 54 HLA-B27 AAU (19) IIU (15) BU (19) HC *n* = 26	miR-140-5p, miR-491-5p, miR-223-3p, miR-223-5p, miR-193a-5p, miR-29a-3p U6 snRNA-uveitis associated cluster	-	Serum	Open Array (TaqMan), RT-qPCR (TaqMan)	Verhagen FH. et al., 2018 (Verhagen et al. 2018)
Patients *n* = 40 BD (10) Sarcoidosis (17) VKH (13) HC *n* = 11	miR-1226-3p, miR-133b, miR-193b-3p, miR-204-5p, miR-2277-5p, miR-326, miR-422a, miR-4652-3p, miR-4698, miR-483-3p, miR-495-5p, miR-519d-5p, miR-520f-5p, miR-542-5p, miR-548ab, miR-6734-3p, miR-6744-3p, miR-6769b-3p	miR-1301-5p, miR-193a-5p, miR-300, miR-302c-5p, miR-3605-5p, miR-3612, -miR-379-5p, miR-3936, miR-3978, -miR-4461, miR-4633-3p, miR-4681, miR-4694-3p, miR-4724-5p, miR-4755-3p, -miR-5190, miR-5192, miR-6501-3p, hsa-miR-6728-5p, miR-6804-5p, miR-6817-5p, miR-6823-5p, miR-6853-5p, miR-7162-3p, miR-758-5p, miR-8060, miR-920	Serum	Microarray (3D-Gene miRNA Labeling kit; 3D-Gene Human miRNA Oligo Chip)	Asakage M, et al. 2020 (Asakage et al. 2020)
Patients n = 15 HLA-B27 + (7) AIU ( 8) HC *n* = 5	miR-146a, miR-155, miR-125a-5p	-	PBMC	TaqMan miRNA Assays	O’Rourke M, et al. 2019 (O’Rourke et al. 2019)
Patients *n* = 21 PPU/S (11) SS (10) HC *n* = 16	miR-455-3p, let-7b-5p, miR-5000-5p, miR-6716-3p, miR-6806-3p, miR-106a-5p, miR-4663, miR-548a-3p, miR-450b-5p, miR-17-5p, let-7c-5p, miR-5189-3p, miR-6716-3p	miR-6805-5p, miR-22-3p, miR-23a-3p, miR-1184, miR-425-5p, miR-3621, miR-4467, miR-191-5p, miR-3613-3p, miR-4668-5p, miR-4674, miR-185-3p, miR-378 h, miR-3197	Serum	Microarray (GeneChip® miRNA 4.0 arrays)	Saito S, et al. 2022 (Saito et al. 2022)
Patients n = 29 PBL (17) IU (12)	miR-155, miR-200, miR-22	-	Vitreous	Quantitive real-time PCR-based miRNA panel (StepOnePlus RT-PCR System)	Tuo J, et al. 2014 (Tuo et al. 2014)
Patients *n* = 62 BD (active – 23, inactive – 19) VKH (21) HC *n* = 20	-	miR-155 in active BD	PBMC Dendritic cells CD4 + cells	miRNA TaqMan assay	Zhou Q, et al. 2012 (Zhou et al. 2012b)
Patients *n* = 56 BD (28) Neuro BD (28) HC *n* = 30	-	miR-185 in BD and neuro BD	PBMC	RT-qPCR (miScript II RT Kit, miScript Primer Assay)	Ugurel E, et al. 2015 (Ugurel et al. 2016)
Patients *n* = 6 BD	miR-4505, miR-149-3p	let-7d-5p, miR-181a-5p, miR-146a-5p, miR-361-5p, miR-532-3p, miR-423-5p, miR-200c-3p, miR-30e-5p, miR-28-5p, miR-30c-5p, miR-330-3p, miR-194-5p, miR-423-3p, miR-28-3p, miR-15b-5p, miR-30d-5p, miR-193a-5p, miR-192-5p, miR-152-3p, miR-25-3p, miR-181d-5p, let-7f-5p, miR-92b-3p, miR-30a-5p, miR-223-3p, miR-505-3p, miR-128-3p, miR-148b-3p, miR-328–3,p miR-195-5p, let-7e-5p, miR-29b-1-5p, miR-628-3p, miR-92a-1-5p, miR-27b-3p, miR-671-3p, miR-151a-3p, miR-486-5p, miR-199a-3p, miR-199b-3p, miR-126-3p miR-584-5p miR-199a-5p miR-139-5p miR-143-3p	PBMC	Microarray (Affymetrix GeneChip® miRNA 4.0) Real-time PCR (TaqMan® Advanced miRNA assay)	Puccetti, A. et al. 2018 (Puccetti et al. 2018)
Patients *n* = 46 BD HC *n* = 26	miR-155		PBMC	Real-time PCR (ABI Prism 7000 Sequence Detection System)	Na SY, et al. 2016 (Na et al. 2016)
Patients *n* = 47 BD HC *n* = 61	miR-155	miR-146a	PBMC	RT-PCR	Kolahi S, et al. 2018 (Kolahi et al. 2018)
Patients *n* = 46 BD HC *n* = 70	-	miR-21	PBMC	qRT-PCR (ExiLENT SYBR Green Master mix)	Jadideslam G, et al. 2019 (Jadideslam et al. 2019)
Patients *n* = 47 BD HC *n* = 34	miR-571, miR-127, miR-573, miR-770, miR-370, miR-139-3p, miR-720, miR-330	miR-200b, miR-520c-3p, miR-199a-3p, miR-224, miR-98	PBMC	Profiling miRnome (Human MicroRNA TaqMan Low-Density Array)	Erre GL, et al. 2015 (Erre et al. 2015)
Patients *n* = 46 BD HC *n* = 48	miR-224-5p, miR-206, miR-653-5p	-	Serum	Microarray (Agilent Human miRNA 8 × 15 k Microarray kit v3.0 and miRNA Complete Labelling and Hyb Kit) PTA-PCR (miRNA-specific primers)	Emmi G, et al. 2022 (Emmi et al. 2022)
Patients *n* = 51 BD HC *n* = 45	miR-181b	-	Serum	RT-PCR (miScript miRNA PCR custom array)	El Boghdady NA, et al. 2019 (Boghdady and Shaker 2019)
Patients *n* = 38 VKH HC *n* = 25	-	miR-20a-5p	PBMC	Quantitive real-time PCR (miDETECT A Track Kit, ABI 7500 System)	Chang R, et al. 2018 (Chang et al. 2018)
Patients *n* = 47 BD HC *n* = 58	miR‐25, miR‐106b, miR‐93, miR‐326	miR-155, miR-146a	PBMC	RT‐PCR (LightCycler 2.0 RT‐PCR System)	Ahmadi M, et al. 2019 (Ahmadi et al. 2019)
Patients *n* = 786 AAU AS + (384) AAU AS- (384) HC *n* = 660	miR-9–3 in high CNV group	miR-9–3, miR-143, miR-146a	PBMC	TaqMan-based qPCR	Yang L, et al. 2017 (Yang et al. 2017)
Patients *n* = 19 Initial onset VKH = 6 Recurrent VKH = 6 HC = 6	miR-206 in both stages of the disease	miR-7704 in recurrent stage	PBMC	NEBNext® Multiplex Small RNA Library Prep Set for Illumina; TruSeq SR Cluster Kit v3-cBot-HS; Illumina Hiseq 2500/2000 platform	Gou K. et al. 2022 (Guo et al. 2022)

#### miRNA in juvenile idiopathic arthritis

There are multiple differences between the selected studies regarding clinical characteristics, type of tissue examined, and miRNA isolation and analysis method. However, changes in the expression of miR-16, miR-125, miR-146a, miR-155, and miR-223 are confirmed. In juvenile arthritis, miR-146a is upregulated in PBMCs, synovial fluid, and serum. The increased expression of miR-146a occurs in response to inflammatory processes. This microRNA promotes the alternative activation of M2 macrophages, associated with regulatory and anti-inflammatory functions. Additionally, miR-146a inhibits the polarization of macrophages towards the M1 phenotype (Ma et al. 2016; Nziza et al. 2019; Li et al. 2016a).

The expression of miR-155 shows inconsistency across different studies. It regulates the immune response pathway by modulating IL-2 and IL-6 expression and can be involved in apoptosis and granulopoiesis. Research by Lashine and colleagues suggests its potential as a biomarker to distinguish lupus-associated arthritis from other inflammatory arthritic conditions. Their results showed reduced miR-155 expression in PBMCs from patients with lupus-associated arthritis and increased expression in individuals with juvenile arthritis (Lashine et al. 2015). MiR-155 was also reported to be elevated in synovial fluid (along with miR-146a and miR-6764-5p) from patients with JIA compared to septic arthritis (SA). Overexpression of these three miRNAs in SF distinguishes JIA from septic arthritis and suggests their role in autoimmunity rather than infectious inflammation (Nziza et al. 2019).

Ma et al. found decreased serum levels of miR-155 in poly-JIA patients compared to controls. Conversely, Demir et al. observed significantly elevated miR-155 levels in both active and inactive JIA. Despite these conflicting findings, both studies agree on the increased expression of miR-16 in JIA patients compared to healthy controls. Furthermore, plasma miR-16 levels have been shown to increase during active JIA and remain elevated in remission (Ma et al. 2016; Demir et al. 2018).

**Fig. 4:**
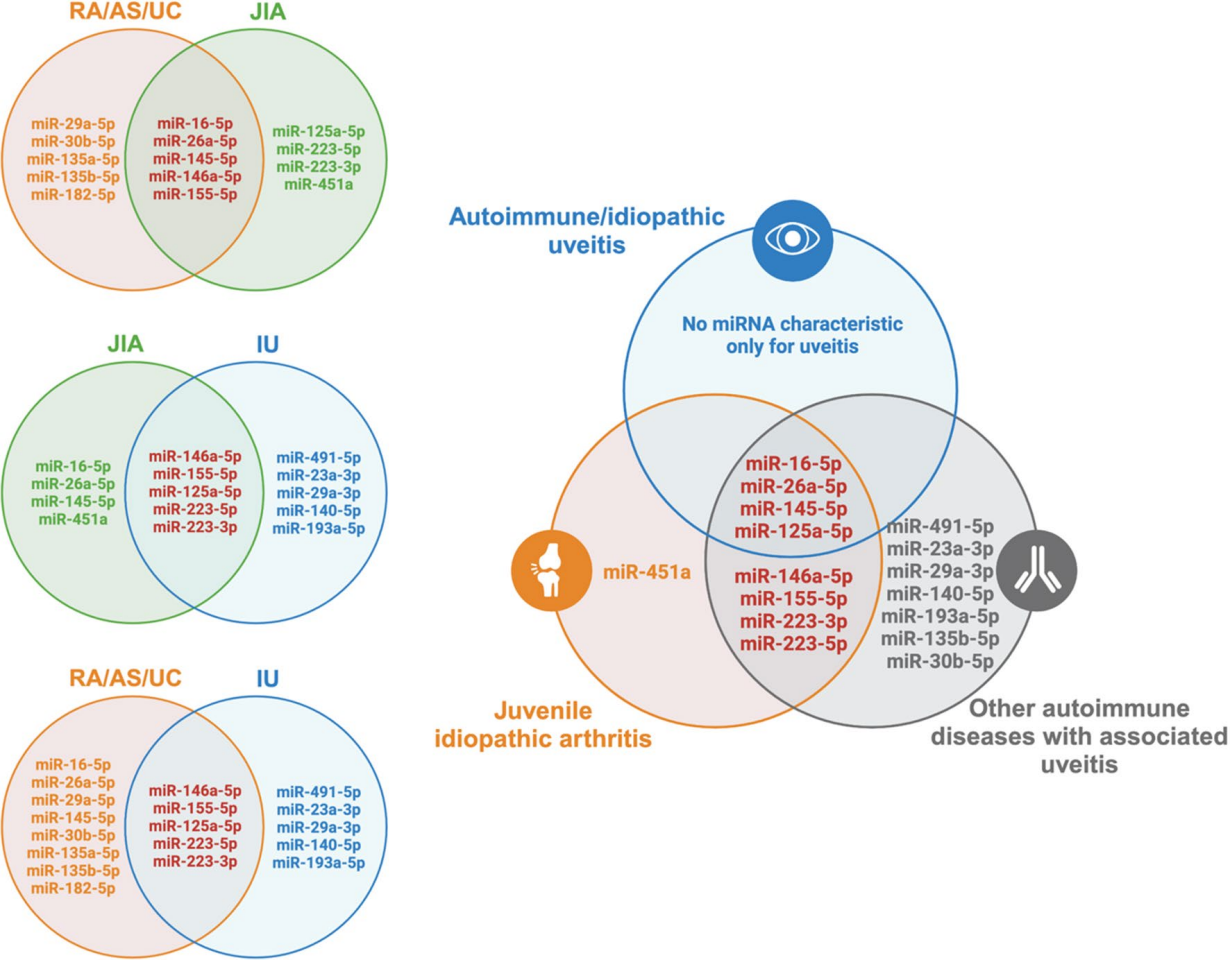
Selected miRNAs involved in uveitis, juvenile idiopathic arthritis, and other autoimmune diseases. Changes in the expression of miR-125a-5p, miR-146a-5p, miR-155-5p, miR-223-3p, and miR-223-5p occur in NIU, JIA, and other autoimmune diseases that often are associated with uveitis (rheumatoid arthritis (RA) (Cunningham et al. 2021; Evangelatos et al. 2019), ankylosing spondylitis (AS) (Motta et al. 2020), ulcerative colitis (UC) (Yarani et al. 2022), lupus erythematosus (LE) (Wang et al. 2010)). There are no miRNAs specific only for uveitis or common only for uveitis and juvenile idiopathic arthritis that are not detected in other autoimmune disorders. Created with BioRender.com

In contrast to miR-16, miR-223 appears to correlate with disease activity. Elevated levels of miR-223 have been observed during active phases of both systemic and polyarticular JIA, suggesting its potential as a biomarker of disease activity (Kamiya et al. 2015). Levels of miR-300, miR-144, miR-133b, and miR-744, which are correlated with leukocyte adherence and transmigration pathways, differ between treated and untreated patient groups. It can be a useful tool in assessing the effectiveness of the treatment (Hu et al. 2018). It is also reported that the miRNA expression profile can change locally to promote joint-restricted inflammatory reactions. miR-23a regulates mitochondrial reactive oxygen species (ROS) release and is overexpressed in synovial fluid mononuclear cells compared to peripheral blood mononuclear cells (Rajendiran et al. 2022). Table 4 summarizes papers on the subject of miRNA in juvenile idiopathic arthritis enrolled in this study. Figure 4 illustrates common and differentiating miRNAs for idiopathic uveitis and other autoimmune conditions.

**Table 4:** miRNA in juvenile idiopathic arthritis. *HC* healthy controls, *JIA* juvenile idiopathic arthritis, *sJIA* systemic JIA, *pJIA* polyarticular JIA, *oJIA* oligoarticular JIA, *JAS* juvenile ankylosing spondylitis, *SLE* systemic lupus erythematosus, *KD* Kawasaki disease, *HSP* Henoch-Schönlein purpura, *SA* septic arthritis, *PBMC* peripheral blood mononuclear cells, *RF* rheumatoid factor

Patients and diagnosis	miRNA upregulated	miRNA downregulated	Sample	miRNA identification method	Reference
Patients *n* = 24 sJIA (8) pJIA (16) HC *n* = 8	miR-223 (different expression between active and inactive phase)	miR-155 in pJIA	Serum	RT-qPCR (TaqMan)	Kamiya Y, et al. 2015 (Kamiya et al. 2015)
Patients *n* = 45 Juvenile SLE ( 25) JRA (10) Juvenile FMF (10) HC *n* = 15	miR-155 in JIA	miR-155 in JSLE	PBMC	RT-qPCR (TaqMan)	Lashine YA, et al. 2015 (Lashine et al. 2015)
Patients *n* = 240 sJIA (40) oJIA (40) pJIA (25) SLE (25) JAS (30) KD (40) HSP (40) HC *n* = 40	miR-26a, miR-145, miR-181a, and miR-1228	miR-1237	Serum	Microarray	Sun J, et al. 2016 (Sun et al. 2016)
Patients *n* = 109 oJIA (43) pJIA (37) JAS (29) HC *n* = 30	miR-146a, miR-16, and miR-223, (miR-16 upregulated pJIA > oJIA)	miR-132, miR-155	Serum	Microarray RT-qPCR (SYBR-Green)	Ma X, et al. 2016 (Ma et al. 2016)
Patients *n* = 20 sJIA HC *n* = 20	-	miR-21, miR-19a	PBMC	RT‑qPCR	Li HW, et al. 2016 (Li et al. 2016b)
Patients *n* = 32 sJIA	miR-146a	-	PBMC	qPCR (TaqMan)	Li D, et al. 2016 (Li et al. 2016a)
Patients *n* = 27 sJIA HC *n* = 15	miR-125a-5p, miR-181a, miR-181c—in active sJIA	-	PBMC	microRNA TaqMan array	Schulert GS, et al. 2016 (Schulert et al. 2016)
Patients *n* = 31 JIA HC *n* = 31	miR-155, miR-16	miR-451, miR-204	Serum	RT-qPCR (SYBR Green)	Demir F, et al. 2018 (Demir et al. 2018)
Patients *n* = 75 JIA: active (35); active + treatment (26); inactive (14) HC *n* = 35	miR-144	miR-744, miR-144, miR-133b, miR-381, miR-300	PBMC	GeneChip (Affymetrix)	Hu Z, et al. 2018 (Hu et al. 2018)
Patients *n* = 26 JIA (13) SA (13)	miR-6764-5p, miR-155, miR-146a-5p in SF JIA > SA	-	Synovial fluid/serum	RT-PCR (TaqMan)	Nziza N, et al. 2019 (Nziza et al. 2019)
Patients *n* = 33 JIA HC *n* = 20	-	miR-21	PBMC	qRT-PCR	Li HW, et al. 2020 (Li and Zeng 2020)
Patients *n* = 16 JIA RF + HC *n* = 22	-	miR-125b	PBMC	qPCR (SYBR Green)	Fan ZD, et al. 2020 (Fan et al. 2020)
Patients *n* = 32 sJIA HC *n* = 21	-	miR-150-5p	PBMC	RT-qPCR (SYBR Green)	Jiang H, et al. 2021 (Jiang et al. 2021)
Patients *n* = 9 oJIA HC *n* = 8	miR-23a (overexpression in SFMCs compared to PMBCs)	-	Synovial fluid mononuclear cells (SFMC) and PBMC	Microarray RT-qPCR (TaqMan)	Rajendiran A, et al. 2022 (Rajendiran et al. 2022)
Patients *n* = 13 oJIA	let-7c-5p, miR-21-5p, miR-34a-5p, miR-125b-5p, miR-155-5p, miR-193b-3p, miR-218-5p	let-7a-5p, let-7e-5p, let-7 g-5p, miR-16-5p, miR-17-5p, miR-20a-5p, miR-26b-5p, miR-106b-5p	Extracellular vesicles/synovial fluid/serum	Array card	Raggi F, et al. 2022 (Raggi et al. 2022)
Patients *n* = 40 JIA HC *n* = 20	miR-15a-5p, miR-409-3p in serum	miR-451a, miR-192-5p	Serum/synovial fluid	ddPCR (EvaGreen)	McAlpine SM, et al. 2023 (McAlpine et al. 2023)
Patients *n* = 8 JIA	miR-27a-3p	-	Synovial fluid	qPCR (SYBR Green)	Bullock CH, et al. 2023 (Bullock et al. 2023)

### Analysis of miRNA targets and signaling pathways

**Fig. 5:**
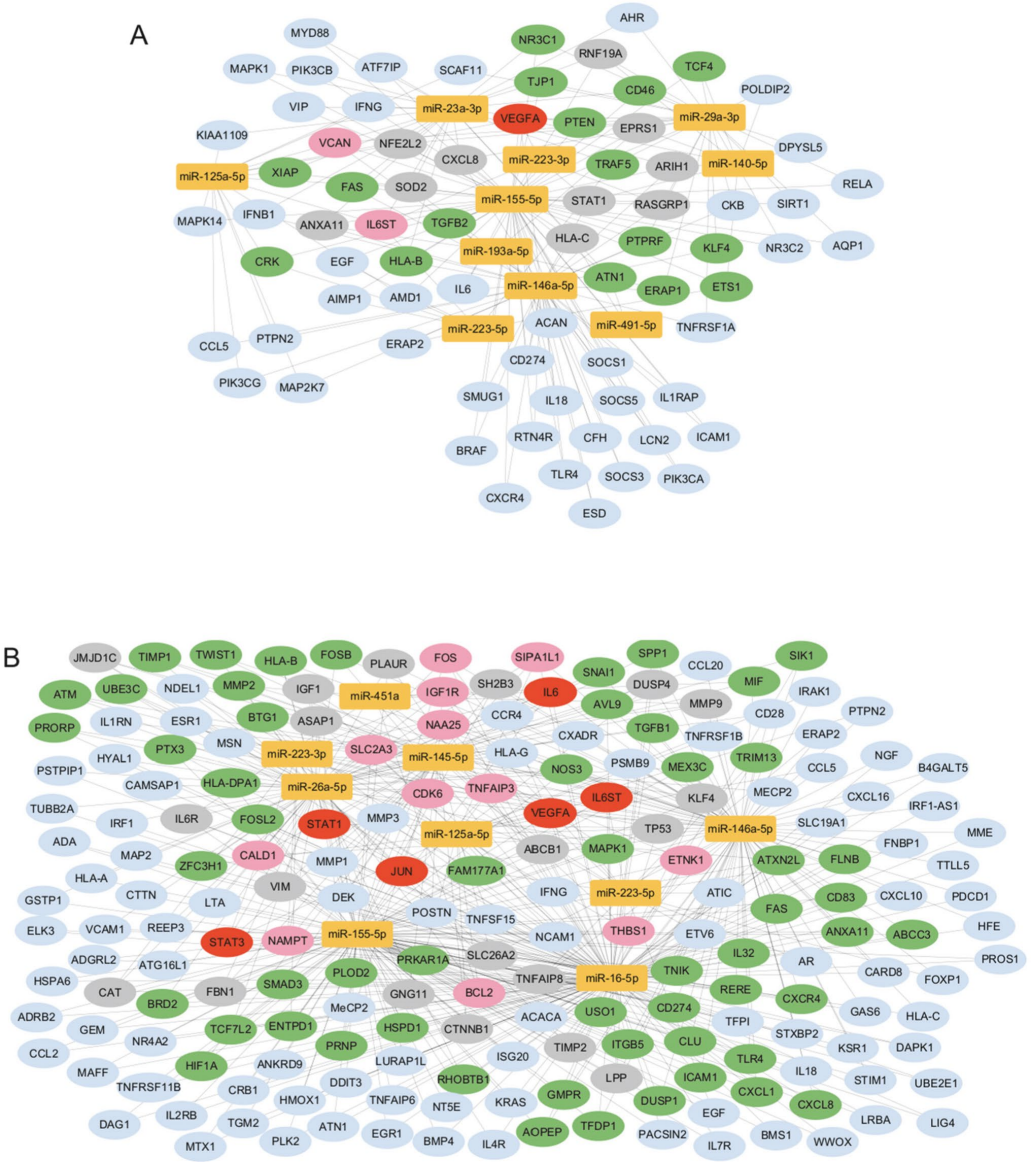
Regulatory network presenting interactions between ten most frequently expressed miRNAs in uveitis (**A**) also nine most frequently expressed miRNAs in juvenile idiopathic arthritis–associated uveitis (**B**) and mRNA targets obtained from DisGeNET 7.0 database as associated with uveitis and juvenile idiopathic arthritis. miRNAs are depicted in yellow circles, and the mRNA targets are depicted in blue (targeted by two miRNAs), green (targeted by three miRNAs), gray (targeted by four miRNAs), pink (targeted by fifth miRNAs), and red (targeted by six miRNAs) circles. Interactions were found in silico using multiMiR 1.26.0 package in R

To recognize a regulatory function of analyzed miRNAs in non-infectious uveitis pathology, we performed in silico target annotation analysis between 10 miRNAs found in patients with uveitis and 247 uveitis-associated genes received from DisGeNET 7.0 database (Concept Unique Identifier “C0042164” was queried); likewise, 9 miRNAs found in patients with juvenile idiopathic arthritis-associated uveitis and 450 juvenile arthritis associated genes received from DisGeNET 7.0 database (Concept Unique Identifier “C3495559” was queried). Target annotation analysis revealed 264 validated miRNA-gene pairs for uveitis, including 142 unique genes (Supplementary Table 4). Of these, 74 genes were regulated by more than one of the identified miRNAs, with 1 gene being target of six of the miRNAs (*VEGFA*), 2 genes being targets of fifth miRNAs (*VCAN*, *IL6ST*), 10 genes being targets of fourth miRNAs, 16 genes being targets of three miRNAs, and 45 genes being targets of two miRNAs. In the case of idiopathic arthritis–associated uveitis, target annotation analysis revealed 648 validated miRNA-gene pairs, including 299 unique genes (Supplementary Table 5). Of these, 193 genes were regulated by more than one of the identified miRNAs, with six genes being targets of six of the miRNAs (*VEGFA*, *IL-6*, *IL6ST*, *JUN*, *STAT1*, *STAT3*), 12 genes being targets of fifth miRNAs, 20 genes being targets of fourth miRNAs, 56 genes being targets of three miRNAs, and 99 genes being targets of two miRNAs. Identified interactions (genes regulated by more than one of the identified miRNAs) were visualized on the regulatory network (Fig. 5).

**Fig. 6:**
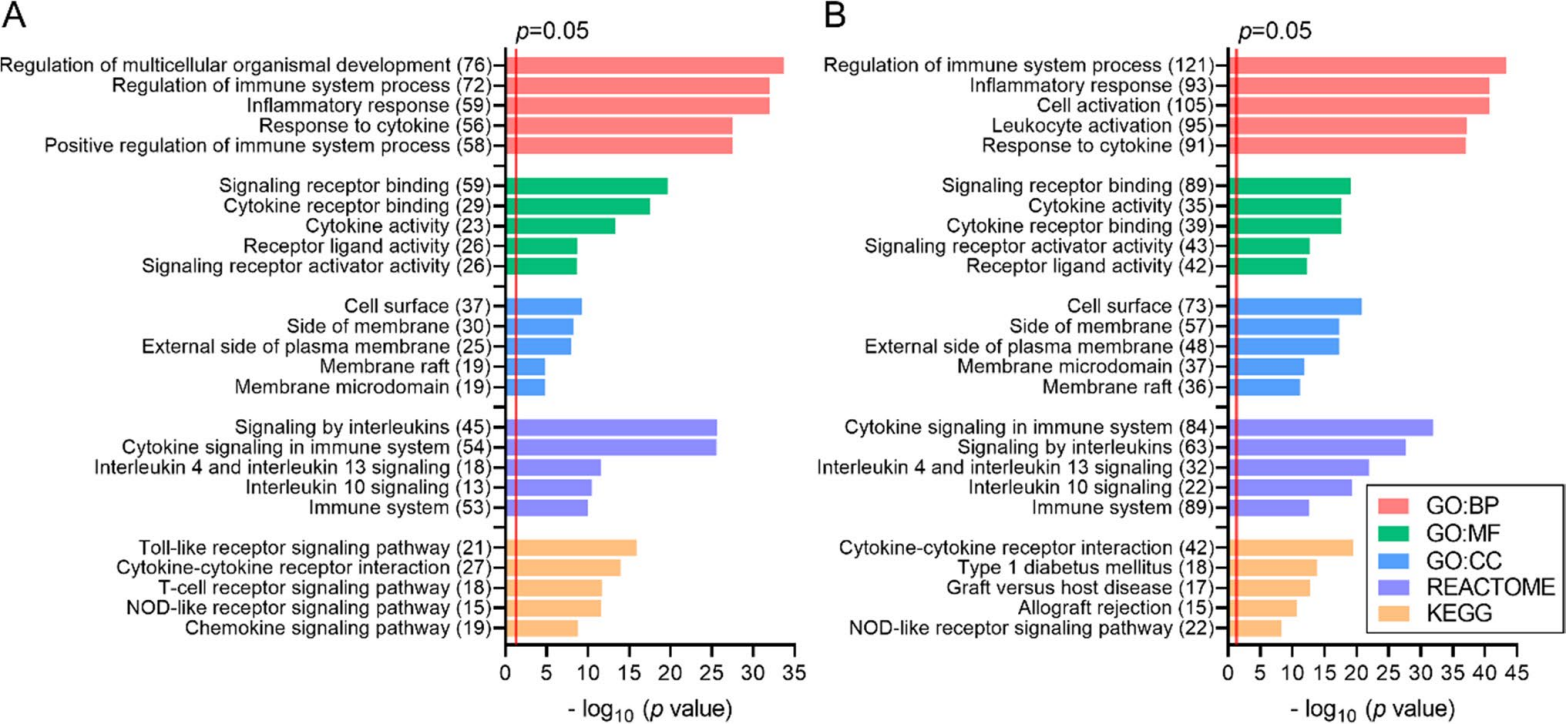
Top five the most enriched terms of GO (Gene Ontology) Biological Processing (GO: BP), GO Molecular Function (GO: MF), GO Cellular Compartment (GO: CC), KEGG (Kyoto Encyclopedia of Genes and Genomes), and REACTOME categories, revealed for uveitis (**A**) and juvenile idiopathic arthritis–associated uveitis (**B**) associated genes targeted by miRNAs found in the current study. *p-value*—EASE score for enrichment adjusted by Benjamini correction for multiple hypothesis testing. The number in brackets following the name of terms indicates the number of associated genes

To indicate biological processes in which miRNA genes are involved, functional analysis was performed for 142 networked genes (uveitis) and 299 networked genes (juvenile idiopathic arthritis–associated uveitis) using the ToppGene Suite website tool (Supplementary Tables 6 and 7). Figure 6 presents the top five most enriched terms of GO (Gene Ontology) Biological Processing, GO Molecular Function, GO Cellular Compartment, KEGG, and REACTOME categories.

Genes found as targets of the most frequently expressed miRNAs in both uveitis and juvenile idiopathic arthritis–associated uveitis were associated with the regulation of immune system process and inflammation, response to cytokines, inflammatory response, cell surface, signaling by interleukins, cytokine-cytokine receptor interactions, Toll-like, T-cell, NOD-like receptor, VEGF, and JAK/STAT signaling pathways.

## Discussion

Non-coding RNAs regulate multiple cellular processes in every organ in the human body. They promote cellular development and maturation as well as pathological processes. Their involvement in immunological pathways is well established, but their role in childhood autoimmune uveitis is poorly understood and needs to be explored. Non-infectious uveitis in children is commonly idiopathic or associated with JIA. The miRNA environment in this rheumatic disease is somewhat better understood than in uveitis, but to our knowledge, there is no research investigating the role of non-coding RNAs in pediatric idiopathic or autoimmune uveitis or exploring the common background of IU and JIA-AU.

In this study, we analyzed the expression profile of a set of microRNAs in the blood of pediatric uveitis patients and supplemented with data obtained from the meta-analysis. We focused on studies conducted on human samples, mainly because we were looking for miRNA that could be a potential biomarker of idiopathic uveitis. Therefore the sample collection should be as non-invasive, repeatable, and simple as possible. Experimental autoimmune uveitis (EAU) tests are often based on eye tissue samples (Wei et al. 2019; Hsu et al. 2015). Although we excluded research on animal models from our meta-analysis, the information on miRNA expression and the course of action that EAU provides are invaluable, especially since the results of miRNA analysis from animal models and human samples are similar and comprise immune-related particles. Several studies have recently reported microRNA expression profiles in adult uveitis patients. However, no one has performed such an analysis on samples from children. Interestingly, our results show similarities with these previous studies but also major differences. Consistent with gene expression studies in adults, a similar miRNA expression pattern in patients with IU may indicate a common molecular basis for this disease with other autoimmune diseases (Rosenbaum et al. 2021a, 2021b). Alterations in miR-30b-5p expression levels are observed in human rheumatoid arthritis and animal models of autoimmune uveitis (Romo-Garcia et al. 2019; Sun et al. 2018). The overexpression of miR-30b-5p and other “inflammatory” miRNAs in patients P8, P13, and P14 may indicate that uveitis precedes joint symptoms and that these patients may develop JIA or RA in the future. Assessing the accuracy of the assumptions made requires long-term patient follow-up and further research on changes in the expression of selected miRNAs. Downregulation of miR-155-5p in the JIA-AU group (0.30 ± 0.04, *p* = 0.0004) and most patients from the IU group (2.01 ± 0.37, *p* = 0.3863) is consistent with literature findings (Fig. 1). Similarly, the diverse expression profile of miR-146a-5p is also supported by other research (see Tables 3 and 4). miR-146a-5p is involved in several immunological pathways and can be up- or downregulated in the serum of patients with autoimmune diseases. Also, the differences between miR-146a-5p expression levels in different papers and our patient group (for JIA-AU patients 0.44 ± 0.04, *p* = 0.0409; for IU patients 1.94 ± 0.18, *p* = 0.2575) (Fig. 1) may occur because the immune response is an active process and is a constant conversation between immune cells through cytokines and transcription factors creating positive and negative control loops. Therefore, although miR-146a-5p is a key regulator of Th17 development, its expression levels vary at different stages of the immune response (Watanabe et al. 2016). At this point, it may not be a perfect candidate for a molecular marker of the disease. In agreement with the previous publication and the known mechanism of its action, miR-204-5p expression is downregulated in both JIA-AU (0.12 ± 0.01, *p* < 0.0001) and IU groups (0.15 ± 0.02, *p* < 0.0001) (Fig. 1) compared to healthy controls (Demir et al. 2018).

Rosenbaum et al. investigated in the adult population the transcriptional signatures from peripheral blood and identified specific subsets among patients with idiopathic uveitis. Eleven out of 38 patients with idiopathic uveitis had their diagnosis revised based on gene expression profiling. This is especially important when the change of the diagnosis results in a different treatment approach or has different prognostic implications (Rosenbaum et al. 2021a). In juvenile idiopathic arthritis, different subtypes correlate differently with the risk of uveitis and the need for regular ophthalmological checkups. Thirty percent of patients with oligoarticular JIA will present with bilateral, insidious, usually asymptomatic uveitis. In contrast, enthesitis-related arthritis is typically associated with acute anterior uveitis with redness and pain. In both subtypes, the inflammation of the uvea may occur before the joint involvement (Zaripova et al. 2021; Sen and Ramanan 2020). The “idiopathic” term is usually used in cases where the patient does not fit the diagnostic criteria. We assume that similar to the adult population, molecular characterization using miRNAs in the pediatric group could provide useful information and allow the reclassification of patients with idiopathic uveitis.

Due to the problem of enrolling a group of patients before any treatment was initiated, we did not correlate the level of inflammatory markers with the selected miRNA expression profiles. Patients with a rheumatological diagnosis had their treatment initiated by rheumatologists, and patients with a diagnosis of idiopathic uveitis also went to the clinic after initial treatment started by an ophthalmologist at the district clinic. Taking this into account, we carried out an analysis of selected miRNAs in the two patient groups and the healthy control group regardless of the current or past treatment.

Li et al., in gene expression profiling in the adult population, revealed four distinct subgroups of uveitis patients. They failed to correlate clinical uveitis diagnoses with this differential gene expression profiling (Li, et al. 2008). However, the fact that upregulated genes in one group were more functionally associated with cell proliferation pathways and in the other with cell apoptosis explains why the same therapeutic approach can lead to different outcomes. For example, a monoclonal antibody against interleukin-17 works well in psoriasis but is unsuccessful in rheumatoid arthritis (Bilal et al. 2018; Dokoupilova et al. 2018). On the other hand, RNA sequencing data show that many forms of uveitis share overlapping mechanisms. Ten upregulated transcripts correlated with immunological pathways and potentially involved in intraocular inflammation were detected in blood samples from patients with different types of uveitis (Rosenbaum et al. 2021b). Identified transcripts include, for example, three out of four myocyte-enhancing factor 2 (MEF2) genes. MEF2 protein family is expressed in most tissues, but MEF2B expression is enriched in B cells and other lymphoid tissue. MEF2A, MEF2C, and MEF2D are involved in photoreceptor development and retinal angiogenesis (Sacilotto et al 2016; Omori et al. 2015). MEF2 family members are targets for miR-223-5p. In chronic myeloid leukemia, repression of miR-223 results in the activation of MEF2C and its downregulation negatively correlates with disease risk (Agatheeswaran et al. 2013; Carrillo et al. 2023). miR-223 involvement in granulopoiesis and inflammatory signaling cascades is also relevant in JIA and uveitis (Verhagen et al. 2018; Kamiya et al. 2015).

The eye is an immunologically privileged organ with a unique immunosuppressive environment. For clear vision, particularly important is to maintain the immune response under control. The pigmented epithelial (PE) cells of the iris, ciliary body and retina, and corneal endothelial (CE) cells participate in maintaining balance in two ways: firstly, as an anatomical barrier, and secondly, by interacting with active CD4 + cells that have penetrated the blood-ocular barrier via cytokines (Mochizuki et al. 2013; Becker and A.G., Davey MP, Rosenbaum JT. 2000). A disturbance in the balance between Th1, Th2, and Th17 lymphocyte activation and differentiation is the basis of autoimmune uveitis. In addition to the role of these cells, γδ T cells are becoming increasingly prominent in research. They secrete inflammatory cytokines in the initial stages of inflammation, but can also influence the activity of Th17 and escalate the pro-inflammatory effect (Zhang et al. 2023; Zhang and Zhang 2023).

Not all Th17 cells are equally pathogenic, and their development and activity are highly sensitive to the inflammatory environment. In chronic inflammation Th17 subpopulation, characterized by the production of IL-17, IFN-γ, granulocyte- and granulocyte–macrophage colony-stimulating factor (G-CSF and GM-CSF), and IL-6, are closely associated with pathogenicity. IL-17 can induce the expression of pro-inflammatory cytokines including IL-1β, IL-6, and TNF-α, and these cytokines in turn enhance the production and strengthen the effects of IL-17, through the activation of STAT3 and the NF-κB pathway. For Th17 cell differentiation from naïve CD4 + T cells, the presence of IL-23 is critical. In contrast, acute inflammation creates an anti-inflammatory environment that suppresses Th17 cell activity, leading to downregulation of IL-17. Importantly, microRNAs miR-146a and miR-155 emerge as key regulators in this complex system, acting to downregulate the pro-inflammatory effects of the IL-17/IL-23 pathway (Zhong et al. 2021; Hirota et al. 2011).

**Fig. 7:**
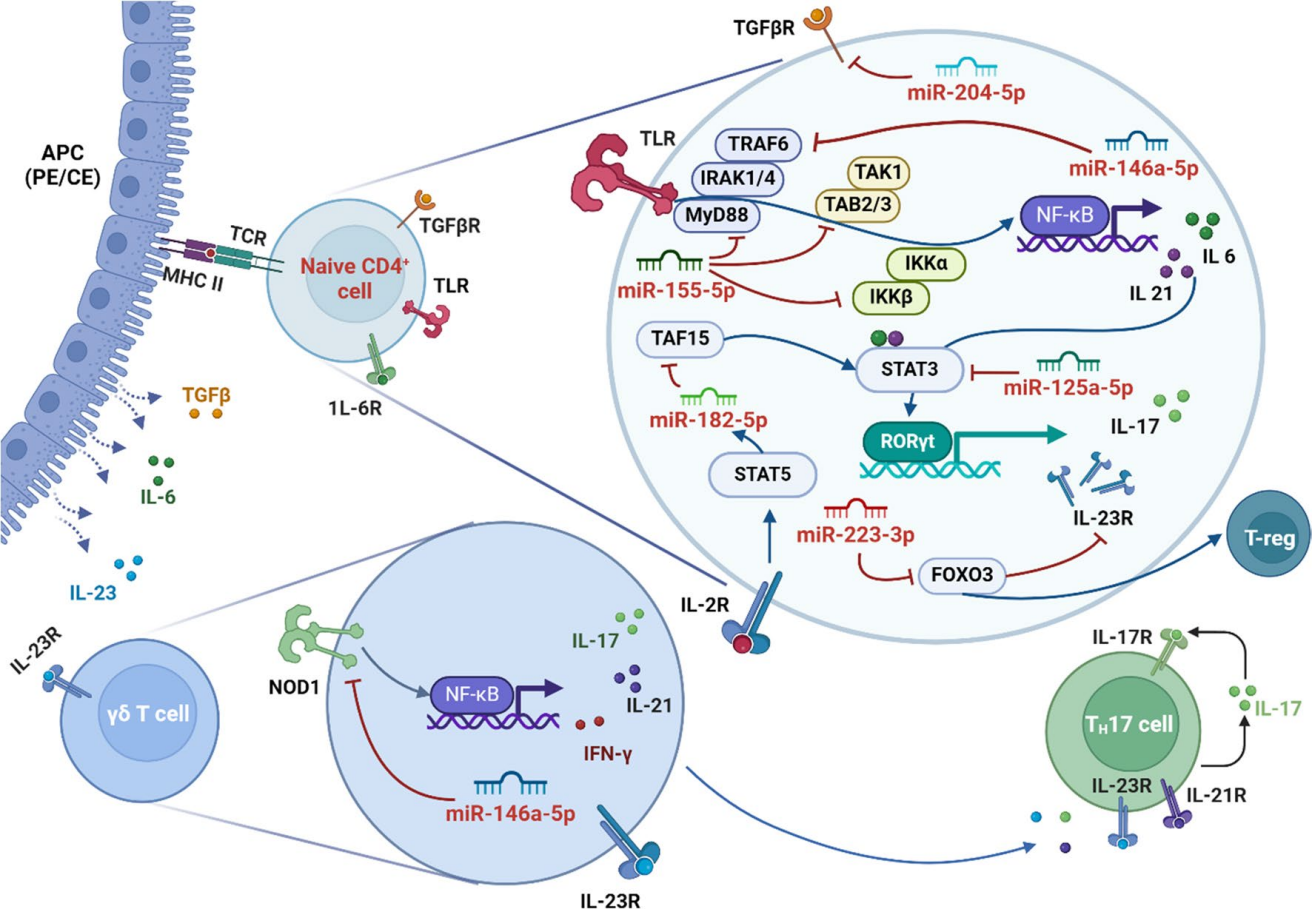
miRNA places of action in T-cell maturation after activation by iris pigmented epithelium (PE) or corneal endothelium (CE) acting as antigen-presenting cells (APC). Blue arrows show activation, and red flat arrows show inhibition. Detailed description of individual miRNAs—see the text. Created with BioRender.com

As shown in Fig. 7, the involvement of miRNA in immunological regulatory pathways occurs at multiple levels and adds complexity to uveitis pathogenesis. The PE and CE cells act as APCs. Through IL-23, they induce naive CD4 + cells to differentiate into Th17 cells, and downregulation of miR-182-5p additionally enhances this process by the transcriptional initiator TATA-binding protein-associated factor 15 (TAF15) upregulation. Overexpression of miR-182-5p by direct binding with TAF15 mRNA can silence its expression and affect STAT3 levels, which lowers IL-23 receptor (IL-23R) levels as well as retinoid-related orphan receptor-gamma t (RORγt) and granulocyte macrophage-colony stimulating factor (MG-CSF) resulting in depressed Th17 activity and immune reaction (Zhang et al. 2020). Forkhead box O3 (FOXO3), a direct target of miR-223-3p, is another transcription factor that negatively regulates IL-23R activity. When miR-223-3p levels are elevated, FOXO3 expression is reduced, resulting in increased IL-23R expression and enhanced Th17 cell activity. This creates a feedback loop where IL-23 signaling upregulates miR-223-3p, further suppressing FOXO3 and perpetuating Th17-mediated inflammation (Wei et al. 2019). miR-125a and miR-204-5p downregulation can result in prompt immune response. miR-125a promotes T-reg development by suppressing T-cell effector factors such as STAT3 and IFN-γ (Pan et al. 2015; Banerjee et al. 2013). miR-205-5p targets the TGF receptors (TGFβR1 and TGFβR2) and reduce the expression of pro-inflammatory cytokines (IL-1, TNF-α) and matrix metalloproteinases (MMP2, MMP9) (Wu et al. 2022; Zhang et al. 2019; Domingues et al. 2016). An intriguing attribute of miRNAs is that a single miRNA can interact with multiple targets. miR-146a-5p is primarily activated by the nuclear factor kappa-light-chain-enhancer of activated B cells (NF-κB). It acts in a negative loop by targeting TNF receptor-associated factor 6 (TRAF6) and interleukin-1 receptor-associated kinase 1 (IRAK1) and silencing NF-κB. As a result, it inhibits IL-6 and IL-21 signals in CD4 + T cells and hampers their differentiation into Th17 cells (Li et al. 2017) (Hsu et al. 2017). In addition, miR-146a-5p regulates Th17 development via IL-2 production and cell death activation (Yang et al. 2012). It also interacts with nucleotide-binding oligomerization domain-containing protein 1 (NOD1) in γδT cells. Nod1 can trigger an inflammatory response that includes the induction of miR-146a. Subsequently, miR-146a directly suppresses NOD1, acting as a cell-intrinsic brake on the ability of γδ27 T cells to produce both IL-17 and IFN-γ, preventing over-activation of the immune response (Schmolka et al. 2018; Cui et al. 2009). Similarly to miR-146a, miR-155 plays a complex role in regulating the immune response. The expression pattern varies between studies, as shown in Tables 3 and 4. It is specific to both tissue type and duration of autoimmune disease (Muhammad et al. 2019). These reflect the fact that miR-155 can manipulate multiple targets in intracellular pathways. In T cells, it interacts with Toll-like receptor (TLR) pathways at different sites, for example, by suppressing myeloid differentiation marker 88 (MyD88), TAK-binding protein (TAB), or receptor-interacting protein (RIP). Reducing the level of miR-155 may contribute to the development of EAAU. To add to the complexity, the pro-inflammatory factor STAT3, controlled indirectly by miR-146a, miR-182-5p, and directly by miR-125a, may also act as a regulator of miR-155, creating an axis promoting the expansion of pathogenic Th17 cells in experimental uveitis (Escobar et al. 2013; Hsu et al. 2015; Hu et al. 2022).

Our results have to be interpreted with the following study limitations in mind. The sample collection process was challenging, as it had to be synchronized with lab accessibility and required an additional 10 ml of blood. The main reason patients and their parents/legal guardians did not agree to participate in the study was reluctance to undergo additional blood collection. Also, during the COVID-19 pandemic, the clinic implemented a restricted admission policy, which complicated patient recruitment and sample collection. As a result, the number of patients included in the study was limited.

Another challenge was the recruitment of a homogenous group of patients. In a tertiary referral center, we observe challenging cases, mainly with chronic or recurrent inflammation resistant to standard treatment and with additional ophthalmological issues. Two of the patients with idiopathic uveitis developed complications, one—secondary glaucoma and one, despite general medications, topical treatment, and extraocular steroid injections—cataract in the left eye. In the JIA group, six out of seven patients were taking a combination of disease-modifying anti-rheumatic drugs (non-biological and biological) at the time of sample collection, and four out of seven had complications such as cataracts or secondary glaucoma.

## Conclusions

Since their discovery, non-coding RNAs such as miRNAs have emerged as master regulators of numerous pathological processes. In the course of idiopathic uveitis, an inflammation of the uvea, many microRNAs show a dysregulated expression profile. As to our knowledge, this is the first published report of miRNA analysis in the blood samples of pediatric patients with uveitis, the data presented in this study bring us closer to an understanding on the implication and contribution of non-coding RNAs in the pathology of uveitis in children. The results provide then a novel perspective regarding the mechanism and diagnosis, especially in the context of difficult-to-diagnose cases of disease. We confirmed that pediatric idiopathic uveitis shares a common molecular basis with other autoimmune diseases at the non-coding RNA level. The analysis of potential targets also confirmed that the miRNAs studied affect key pathways in T-cell regulation and autoimmunity. Importantly, we showed changes in immune-related miRNA expression levels in patients with negative rheumatological history and negative ANA antibodies and RF factor. This suggests that alterations at the molecular level are more sensitive and may occur earlier than changes in “classical” antibody levels. Although further research is needed to validate and expand these findings and advance the clinical integration of miRNA as biomarkers for pediatric patients with uveitis, the diagnostic potential seems to be indisputable. This may lead to uncover novel and still unexplored diagnostic and therapeutic strategies for the management of pediatric uveitis. Confirming the common miRNA expression profile between idiopathic and rheumatological patients may not yet have a clinical impact. As the saying “today’s science is tomorrow’s medicine” seems to be true, the collected information can provide a useful tool to revise the diagnosis of idiopathic uveitis. Differentially regulated miRNA might be then potentially considered the additional diagnostic markers, especially in the difficult diagnostic cases. Development of the diagnostic based on the miRNA together with the antibody profile could make the diagnosis much easier in order to confirm or even change it. On the other hand, isolating a miRNA specific to uveitis can be used as a treatment target, for example, for siRNA, which will be more effective, with fewer side effects than currently available drugs.

## Supplementary Information

Below is the link to the electronic supplementary material.

Supplementary file1 (PDF 367 KB)

## Data Availability

The data that support the findings of this study are available from the corresponding authors upon reasonable request.

## Abbreviations

NIU: Non-infectious uveitis
JIA: Juvenile idiopathic arthritis
JAS: Juvenile ankylosing spondylitis
JSLE: Juvenile systemic lupus erythematosus
AIU: Autoimmune uveitis
BD: Behçet’s disease
RA: Rheumatoid arthritis
UC: Ulcerative colitis
SLE: Systemic lupus erythematosus
