# Supplementary material for: Exploring microRNA signatures in pediatric non-infectious uveitis: meta-analysis and molecular profiling of patient samples
Source: J Appl Genet. 2024 Dec 19;66(3):579–600. doi: 10.1007/s13353-024-00922-8 (PMC12367970; doi:10.1007/s13353-024-00922-8)
Supplement: Supplementary file 1 — Supplementary file1 (PDF 367 KB) [file 13353_2024_922_MOESM1_ESM.pdf]

Exploring MicroRNA Signatures in Pediatric Noninfectious Uveitis: Meta-Analysis and Molecular Profiling of Patient Samples

Olga Wawrzyniak[1], Dariusz Wawrzyniak[2], Michał Smuszkiewicz[2], Paweł Głodowicz[2], Anna Gotz-Więckowska[1,*] and Katarzyna Rolle[2,*]

[1] Department of Ophthalmology, Poznan University of Medical Sciences, A. Szamarzewskiego 84, 61-848 Poznan, Poland
[2] Department of Molecular Neurooncology, Institute of Bioorganic Chemistry Polish Academy of Sciences, Z. Noskowskiego 12/14, 61-704 Poznan, Poland

SUPPLEMENTARY MATERIAL

Supplementary Table 1. Clinical characteristic of patients.

Patient and diagnosis	Age at the diagnosis	Age at sample collection	Sex	ANA	HLA-B27	Additional information /complications	Treatment
P1 (JIA-AU)	4	10	M	+	N/A	-	topical medications
P2 (JIA-AU)	3	17	F	+	N/A	AIOL left eye	topical medications MTX, metyloprednizolon,
P3 (JIA-AU)	7	13	M	+	-	AIOL both eyes	topical medications, adalimumab
P4 (JIA-AU)	4	10	F	+	N/A	macular edema	topical medications, MTX
P5 (JIA-AU)	12	17	M	+	N/A	secondary glaucoma	topical medications, MTX, cyclosporin
P6 (JIA-AU)	3	17	F	+	N/A	-	topical medications, MTX, etanercept, adalimumab
P7 (JIA-AU)	12	14	F	+	+	-	topical medications adalimumab, tofacytynib
P8 (IU)	12	12	F	-	-	-	topical medications
P9 (IU)	16	17	M	N/A	N/A	-	topical medications
P10 (IU)	8	17	M	-	-	AIOL left eye	topical medications, prednizon
P11 (IU)	15	15	F	-	-	-	topical medications
P12 (IU)	10	13	M	+	-	-	topical medications
P13 (IU)	12	14	M	-	-	secondary glaucoma	topical medications
P14 (IU)	16	17	F	N/A	N/A	-	topical medications
P15 (IU)	13	14	M	N/A	N/A	-	topical medications

JIA-AU - juvenile idiopathic arthritis-associated uveitis, IU - Idiopathic uveitis, +/- - positive/negative ANA or HLA-B27 status, MTX – methotrexate, AIOL - artificial intraocular lens, M - male, F - female.

Supplementary Table 2. List of primers used in the study.

DNA Oligo ID	Sequence 5' --> 3'
hsa-ACTN1_F	AATGGGACCACGTGCGGCAG
hsa-ACTN1_R	GGCCTGGGCTCCAAACTGCT
hsa-GAPDH_F	AAGGTGAAGGTCGGAGTCAAC
hsa-GAPDH_R	GGGGTCATTGATGGCAACAATA
hsa-HPRT_F	CGAGCAAGACGTTCAGTCCT
hsa-HPRT_R	TGACCTTGATTTATTTTGCATACC
hsa-SDHA_F	CCTTTCTGAGGCAGGGTTTA
hsa-SDHA_R	ATTTTCTAGCTGTGCTGCAA
hsa-miR-125a-5p	TCCCTGAGACCCTTTAACCTGTGA
hsa-miR-135a-5p	TATGGCTTTTTATTCCTATGTGA
hsa-miR-135b-5p	TATGGCTTTTCATTCCTATGTGA
hsa-miR-140-5p	CAGTGGTTTTACCCTATGGTAG
hsa-miR-145-5p	GTCCAGTTTTCCCAGGAATCCCT
hsa-miR-146a-5p	TGAGAACTGAATTCCATGGGTT
hsa-miR-155-5p	TTAATGCTAATCGTGATAGGGGTT
hsa-miR-16-5p	TAGCAGCACGTAAATATTGGCG
hsa-miR-182-5p	TTTGGCAATGGTAGAACTCACACT
hsa-miR-193a-5p	TGGGTCTTTGCGGGCGAGATGA
hsa-miR-204-5p	TTCCCTTTGTCATCCTATGCCT
hsa-miR-223-3p	TGTCAGTTTGTCAAATACCCCA
hsa-miR-223-5p	CGTGTATTTGACAAGCTGAGTT
hsa-miR-23a-3p	ATCACATTGCCAGGGATTTCC
hsa-miR-26a-5p	TTCAAGTAATCCAGGATAGGCT
hsa-miR-29a-3p	TAGCACCATCTGAAATCGGTTA
hsa-miR-30b-5p	TGTAAACATCCTACACTCAGCT
hsa-miR-451a	AAACCGTTACCATTACTGAGTT
hsa-miR-491-5p	AGTGGGGAACCCTTCCATGAGG

Supplementary Table 3. List of significant outliers. Data not shown on Figure 2.

Patient	miRNA	Value	Outlier
P6 (JIA-AU)	hsa-miR-125a-5p	3.899025961	significant outlier, $p < 0.05$
P6 (JIA-AU)	hsa-miR-135a-5p	1.777946443	significant outlier, $p < 0.05$
P11 (IU)	hsa-miR-135a-5p	11.46581015	significant outlier, $p < 0.05$
P6 (JIA-AU)	hsa-miR-135b-5p	5.460067739	significant outlier, $p < 0.05$
P6 (JIA-AU)	hsa-miR-140-5p	6.5875408	significant outlier, $p < 0.05$
P14 (IU)	hsa-miR-140-5p	3.424429095	significant outlier, $p < 0.05$
P6 (JIA-AU)	hsa-miR-145-5p	4.590271712	significant outlier, $p < 0.05$
P6 (JIA-AU)	hsa-miR-146a-5p	7.117458985	significant outlier, $p < 0.05$
P6 (JIA-AU)	hsa-miR-155-5p	1.881145444	significant outlier, $p < 0.05$
P6 (JIA-AU)	hsa-miR-16-5p	11.2795469	significant outlier, $p < 0.05$
P14 (IU)	hsa-miR-16-5p	5.413939466	significant outlier, $p < 0.05$
P7 (JIA-AU)	hsa-miR-204-5p	377.581661	significant outlier, $p < 0.05$
P6 (JIA-AU)	hsa-miR-223-3p	8.572198199	significant outlier, $p < 0.05$
P8 (IU)	hsa-miR-223-3p	13.00512346	significant outlier, $p < 0.05$
P7 (JIA-AU)	hsa-miR-223-5p	16.31353899	significant outlier, $p < 0.05$
P6 (JIA-AU)	hsa-miR-23a-3p	9.005919806	significant outlier, $p < 0.05$
P6 (JIA-AU)	hsa-miR-26a-5p	12.01046947	significant outlier, $p < 0.05$
P6 (JIA-AU)	hsa-miR-29a-3p	9.272236404	significant outlier, $p < 0.05$
P11 (IU)	hsa-miR-29a-3p	110.8586548	significant outlier, $p < 0.05$
P7 (JIA-AU)	hsa-miR-30b-5p	71.17468258	significant outlier, $p < 0.05$
P11 (IU)	hsa-miR-30b-5p	225.1046426	significant outlier, $p < 0.05$
P6 (JIA-AU)	hsa-miR-451a	18.54736114	significant outlier, $p < 0.05$
P6 (JIA-AU)	hsa-miR-491-5p	7.277495253	significant outlier, $p < 0.05$

Supplementary Table 4. List of genes (193) regulated by more than one of the identified miRNAs in patients juvenile arthritis-associated uveitis (541 miRNA-gene interaction pairs).

Source	Target
miR-16-5p	BCL2
miR-16-5p	VEGFA
miR-16-5p	CARD8
miR-16-5p	JUN
miR-16-5p	CDK6
miR-155-5p	MeCP2
miR-125a-5p	TP53
miR-146a-5p	IRAK1
miR-146a-5p	CXCR4
miR-146a-5p	STAT1
miR-451a	ABCB1
miR-451a	MIF
miR-26a-5p	CDK6
miR-26a-5p	HSPD1
miR-26a-5p	ESR1
miR-26a-5p	ATM
miR-26a-5p	RHOBTB1
miR-26a-5p	SLC26A2
miR-26a-5p	IL6
miR-26a-5p	PLOD2
miR-26a-5p	NAMPT
miR-26a-5p	FAM177A1
miR-26a-5p	IGF1
miR-26a-5p	DUSP4
miR-145-5p	CDK6
miR-145-5p	ESR1
miR-145-5p	VEGFA
miR-145-5p	HIF1A
miR-145-5p	KLF4
miR-145-5p	MMP1
miR-145-5p	STAT1
miR-145-5p	BTG1
miR-145-5p	CD28
miR-145-5p	SLC26A2
miR-145-5p	IGF1R
miR-145-5p	SMAD3
miR-145-5p	DEK
miR-16-5p	HSPD1
miR-16-5p	KRAS
miR-16-5p	CLU
miR-16-5p	VIM
miR-16-5p	CD274
miR-16-5p	TP53
miR-16-5p	ENTPD1
miR-16-5p	HMOX1
miR-16-5p	IFNG
miR-16-5p	IGF1R
miR-16-5p	LIG4
miR-16-5p	SMAD3
miR-16-5p	PLAUR
miR-16-5p	PRKAR1A
miR-16-5p	PRNP
miR-16-5p	SLC2A3
miR-16-5p	TFPI
miR-16-5p	UBE3C
miR-16-5p	BMS1
miR-16-5p	NAMPT
miR-16-5p	ATXN2L
miR-16-5p	FNBP1
miR-16-5p	AVL9
miR-16-5p	TTLL5
miR-16-5p	ETNK1
miR-16-5p	NAA25
miR-16-5p	SIK1
miR-16-5p	CAMSAP1
miR-16-5p	LURAP1L
miR-16-5p	TUBB2A
miR-16-5p	SLC19A1
miR-16-5p	PDCD1
miR-223-3p	ABCB1
miR-223-3p	TP53
miR-223-3p	STAT1
miR-223-3p	ATM
miR-223-3p	STAT3
miR-223-3p	IGF1R
miR-223-3p	IL6
miR-223-3p	IL6ST
miR-223-3p	NAMPT
miR-155-5p	THBS1
miR-155-5p	KRAS
miR-155-5p	VCAM1
miR-155-5p	JUN
miR-155-5p	HIF1A

miR-155-5p	FOS
miR-155-5p	ICAM1
miR-155-5p	STAT1
miR-155-5p	STAT3
miR-155-5p	CAT
miR-155-5p	ENTPD1
miR-155-5p	CTNNB1
miR-155-5p	DAG1
miR-155-5p	FLNB
miR-155-5p	IL6
miR-155-5p	ITGB5
miR-155-5p	SMAD3
miR-155-5p	NOS3
miR-155-5p	NT5E
miR-155-5p	PLAUR
miR-155-5p	PRKAR1A
miR-155-5p	CCL2
miR-155-5p	STIM1
miR-155-5p	STXBP2
miR-155-5p	TFPI
miR-155-5p	TGM2
miR-155-5p	DEK
miR-155-5p	NAMPT
miR-155-5p	PACSIN2
miR-155-5p	MEX3C
miR-155-5p	NAA25
miR-155-5p	FAM177A1
miR-155-5p	HLA-DPA1
miR-155-5p	CXCL8
miR-125a-5p	BCL2
miR-125a-5p	VEGFA
miR-125a-5p	IL1RN
miR-125a-5p	STAT3
miR-125a-5p	IFNG
miR-125a-5p	CCL5
miR-125a-5p	TNFAIP3
miR-125a-5p	NDEL1
miR-146a-5p	FAS
miR-146a-5p	TLR4
miR-146a-5p	TGFB1
miR-146a-5p	FOS
miR-146a-5p	ICAM1
miR-146a-5p	MIF
miR-146a-5p	DUSP1
miR-146a-5p	IL6
miR-146a-5p	PLAUR
miR-146a-5p	CCL5
miR-146a-5p	SPP1
miR-146a-5p	AVL9
miR-146a-5p	CXCL8
miR-451a	BCL2
miR-451a	IL6R
miR-451a	MMP9
miR-451a	IL6
miR-451a	MMP2
miR-451a	MAPK1
miR-223-5p	ETV6
miR-223-5p	FOXP1
miR-223-5p	AR
miR-223-5p	IGF1R
miR-223-5p	IL6ST
miR-223-5p	LPP
miR-223-5p	TRIM13
miR-223-5p	NAA25
miR-223-5p	FAM177A1
miR-223-5p	IGF1
miR-223-5p	TNFAIP8
miR-223-5p	EGF
miR-26a-5p	VCAM1
miR-26a-5p	FBN1
miR-26a-5p	JUN
miR-26a-5p	VIM
miR-26a-5p	FOS
miR-26a-5p	STAT1
miR-26a-5p	STAT3
miR-26a-5p	BTG1
miR-26a-5p	CALD1
miR-26a-5p	CAT
miR-26a-5p	CCR4
miR-26a-5p	CXADR
miR-26a-5p	CTTN
miR-26a-5p	GSTP1
miR-26a-5p	HLA-A
miR-26a-5p	HLA-G
miR-26a-5p	IGF1R
miR-26a-5p	IL6ST
miR-26a-5p	IRF1
miR-26a-5p	MAP2
miR-26a-5p	NOS3
miR-26a-5p	PLAUR
miR-26a-5p	PTX3
miR-26a-5p	SLC2A3

miR-26a-5p	SNAI1	miR-145-5p	PRNP
miR-26a-5p	TIMP1	miR-145-5p	CCL20
miR-26a-5p	TIMP2	miR-145-5p	SNAI1
miR-26a-5p	UBE3C	miR-145-5p	SPP1
miR-26a-5p	AVL9	miR-145-5p	TIMP1
miR-26a-5p	ASAP1	miR-145-5p	TNFAIP3
miR-26a-5p	MEX3C	miR-145-5p	SH2B3
miR-26a-5p	ETNK1	miR-145-5p	NAMPT
miR-26a-5p	NAA25	miR-145-5p	ETNK1
miR-26a-5p	NDEL1	miR-145-5p	TWIST1
miR-26a-5p	SIK1	miR-145-5p	POSTN
miR-26a-5p	CAMSAP1	miR-145-5p	GNG11
miR-26a-5p	ZFC3H1	miR-145-5p	JMJD1C
miR-26a-5p	REEP3	miR-145-5p	SIPA1L1
miR-26a-5p	BRD2	miR-16-5p	THBS1
miR-26a-5p	TWIST1	miR-16-5p	MTX1
miR-26a-5p	LTA	miR-16-5p	FAS
miR-26a-5p	TUBB2A	miR-16-5p	IL6R
miR-26a-5p	FOSB	miR-16-5p	RERE
miR-26a-5p	TCF7L2	miR-16-5p	FBN1
miR-26a-5p	ATG16L1	miR-16-5p	TLR4
miR-26a-5p	HLA-DPA1	miR-16-5p	CD83
miR-26a-5p	GNG11	miR-16-5p	ABCB1
miR-26a-5p	JMJD1C	miR-16-5p	HIF1A
miR-26a-5p	FOSL2	miR-16-5p	TGFB1
miR-26a-5p	HLA-B	miR-16-5p	CXCR4
miR-26a-5p	SIPA1L1	miR-16-5p	MMP3
miR-26a-5p	HYAL1	miR-16-5p	ELK3
miR-26a-5p	PSTPIP1	miR-16-5p	FOS
miR-26a-5p	MIF	miR-16-5p	NR4A2
miR-26a-5p	PRORP	miR-16-5p	ICAM1
miR-145-5p	THBS1	miR-16-5p	KLF4
miR-145-5p	IL6R	miR-16-5p	ETV6
miR-145-5p	FBN1	miR-16-5p	STAT1
miR-145-5p	JUN	miR-16-5p	NCAM1
miR-145-5p	IL1RN	miR-16-5p	ANXA11
miR-145-5p	VIM	miR-16-5p	RHOBTB1
miR-145-5p	TGFB1	miR-16-5p	STAT3
miR-145-5p	MMP3	miR-16-5p	ADA
miR-145-5p	MMP9	miR-16-5p	AR
miR-145-5p	ATM	miR-16-5p	BTG1
miR-145-5p	STAT3	miR-16-5p	CALD1
miR-145-5p	CALD1	miR-16-5p	CAT
miR-145-5p	CTNNB1	miR-16-5p	CTNNB1
miR-145-5p	MMP2	miR-16-5p	DAG1
miR-145-5p	MSN	miR-16-5p	DAPK1

miR-16-5p	DDIT3
miR-16-5p	DUSP1
miR-16-5p	EGR1
miR-16-5p	FLNB
miR-16-5p	GEM
miR-16-5p	CXCL1
miR-16-5p	GSTP1
miR-16-5p	HLA-A
miR-16-5p	IL6
miR-16-5p	IL6ST
miR-16-5p	IL7R
miR-16-5p	IRF1
miR-16-5p	ISG20
miR-16-5p	ITGB5
miR-16-5p	LPP
miR-16-5p	MAP2
miR-16-5p	MME
miR-16-5p	MSN
miR-16-5p	NT5E
miR-16-5p	TNFRSF11B
miR-16-5p	PLOD2
miR-16-5p	MAPK1
miR-16-5p	PROS1
miR-16-5p	PTX3
miR-16-5p	CCL2
miR-16-5p	SPP1
miR-16-5p	TGM2
miR-16-5p	TIMP2
miR-16-5p	TNFAIP3
miR-16-5p	UBE2E1
miR-16-5p	USO1
miR-16-5p	ABCC3
miR-16-5p	IL32
miR-16-5p	B4GALT5
miR-16-5p	TNFSF15
miR-16-5p	SH2B3
miR-16-5p	TRIM13
miR-16-5p	PLK2
miR-16-5p	PACSIN2
miR-16-5p	TNIK
miR-16-5p	CRB1
miR-16-5p	MAFF
miR-16-5p	ASAP1
miR-16-5p	CXCL16
miR-16-5p	ANKRD9
miR-16-5p	ZFC3H1

miR-16-5p	REEP3
miR-16-5p	BRD2
miR-16-5p	CXCL10
miR-16-5p	IGF1
miR-16-5p	PSMB9
miR-16-5p	ADRB2
miR-16-5p	HFE
miR-16-5p	IL4R
miR-16-5p	IL2RB
miR-16-5p	ATN1
miR-16-5p	TNFAIP8
miR-16-5p	FOSB
miR-16-5p	TCF7L2
miR-16-5p	LRBA
miR-16-5p	BMP4
miR-16-5p	DUSP4
miR-16-5p	HSPA6
miR-16-5p	NGF
miR-16-5p	ATIC
miR-16-5p	GNG11
miR-16-5p	JMJD1C
miR-16-5p	FOSL2
miR-16-5p	GMPR
miR-16-5p	TFDP1
miR-16-5p	TNFAIP6
miR-16-5p	SIPA1L1
miR-16-5p	WWOX
miR-16-5p	HYAL1
miR-16-5p	PSTPIP1
miR-16-5p	CXCL8
miR-16-5p	ADGRL2
miR-16-5p	ACACA
miR-16-5p	AOPEP
miR-16-5p	PRORP
miR-16-5p	IRF1-AS1
miR-223-3p	BCL2
miR-223-3p	VEGFA
miR-223-3p	MMP9
miR-223-3p	CAT
miR-223-3p	ENTPD1
miR-223-3p	MECP2
miR-223-3p	MMP2
miR-155-5p	BCL2
miR-155-5p	CDK6
miR-155-5p	HSPD1
miR-155-5p	MTX1

miR-155-5p	VEGFA
miR-155-5p	FAS
miR-155-5p	IL6R
miR-155-5p	RERE
miR-155-5p	FBN1
miR-155-5p	TLR4
miR-155-5p	CD83
miR-155-5p	CLU
miR-155-5p	ABCB1
miR-155-5p	VIM
miR-155-5p	CD274
miR-155-5p	CXCR4
miR-155-5p	TP53
miR-155-5p	ELK3
miR-155-5p	NR4A2
miR-155-5p	KLF4
miR-155-5p	MMP1
miR-155-5p	ANXA11
miR-155-5p	RHOBTB1
miR-155-5p	ADA
miR-155-5p	CALD1
miR-155-5p	DAPK1
miR-155-5p	DDIT3
miR-155-5p	SLC26A2
miR-155-5p	DUSP1
miR-155-5p	EGR1
miR-155-5p	CTTN
miR-155-5p	GAS6
miR-155-5p	GEM
miR-155-5p	CXCL1
miR-155-5p	HMOX1
miR-155-5p	IL6ST
miR-155-5p	IL7R
miR-155-5p	IL18
miR-155-5p	ISG20
miR-155-5p	LIG4
miR-155-5p	LPP
miR-155-5p	TNFRSF11B
miR-155-5p	PLOD2
miR-155-5p	MAPK1
miR-155-5p	PRNP
miR-155-5p	PROS1
miR-155-5p	PTX3
miR-155-5p	SLC2A3
miR-155-5p	TIMP2
miR-155-5p	TNFAIP3

miR-155-5p	UBE2E1
miR-155-5p	USO1
miR-155-5p	ABCC3
miR-155-5p	IL32
miR-155-5p	B4GALT5
miR-155-5p	BMS1
miR-155-5p	SH2B3
miR-155-5p	PLK2
miR-155-5p	ATXN2L
miR-155-5p	TNIK
miR-155-5p	CRB1
miR-155-5p	MAFF
miR-155-5p	ASAP1
miR-155-5p	ETNK1
miR-155-5p	ANKRD9
miR-155-5p	LURAP1L
miR-155-5p	BRD2
miR-155-5p	IGF1
miR-155-5p	ADRB2
miR-155-5p	IL4R
miR-155-5p	LTA
miR-155-5p	IL2RB
miR-155-5p	ATN1
miR-155-5p	TNFAIP8
miR-155-5p	POSTN
miR-155-5p	TCF7L2
miR-155-5p	EGF
miR-155-5p	ATG16L1
miR-155-5p	LRBA
miR-155-5p	BMP4
miR-155-5p	HLA-C
miR-155-5p	HSPA6
miR-155-5p	GNG11
miR-155-5p	FOSL2
miR-155-5p	HLA-B
miR-155-5p	GMPR
miR-155-5p	TFDP1
miR-155-5p	TNFAIP6
miR-155-5p	KSR1
miR-155-5p	WWOX
miR-155-5p	ADGRL2
miR-155-5p	ACACA
miR-155-5p	AOPEP
miR-125a-5p	THBS1
miR-125a-5p	JUN
miR-125a-5p	IRAK1

miR-125a-5p	NCAM1
miR-125a-5p	CTNNB1
miR-125a-5p	IL6ST
miR-125a-5p	PRKAR1A
miR-125a-5p	UBE3C
miR-125a-5p	TNFSF15
miR-125a-5p	ATXN2L
miR-125a-5p	ZFC3H1
miR-125a-5p	TNFRSF1B
miR-125a-5p	PTPN2
miR-125a-5p	DUSP4
miR-125a-5p	SIPA1L1
miR-146a-5p	CDK6
miR-146a-5p	THBS1
miR-146a-5p	VEGFA
miR-146a-5p	RERE
miR-146a-5p	CD83
miR-146a-5p	CARD8
miR-146a-5p	JUN
miR-146a-5p	CLU
miR-146a-5p	CD274
miR-146a-5p	MMP9
miR-146a-5p	KLF4
miR-146a-5p	FOXP1
miR-146a-5p	ANXA11
miR-146a-5p	CALD1
miR-146a-5p	CD28
miR-146a-5p	CCR4
miR-146a-5p	CXADR
miR-146a-5p	SLC26A2
miR-146a-5p	FLNB
miR-146a-5p	GAS6
miR-146a-5p	CXCL1
miR-146a-5p	HLA-G
miR-146a-5p	IL18
miR-146a-5p	ITGB5
miR-146a-5p	LPP
miR-146a-5p	MECP2
miR-146a-5p	MME
miR-146a-5p	NOS3
miR-146a-5p	CCL20
miR-146a-5p	SLC2A3
miR-146a-5p	SNAI1
miR-146a-5p	STIM1
miR-146a-5p	STXBP2

miR-146a-5p	TIMP1
miR-146a-5p	TIMP2
miR-146a-5p	TNFAIP3
miR-146a-5p	USO1
miR-146a-5p	ABCC3
miR-146a-5p	IL32
miR-146a-5p	SH2B3
miR-146a-5p	TRIM13
miR-146a-5p	TNIK
miR-146a-5p	FNBP1
miR-146a-5p	TTLL5
miR-146a-5p	ASAP1
miR-146a-5p	MEX3C
miR-146a-5p	ETNK1
miR-146a-5p	CXCL16
miR-146a-5p	ERAP2
miR-146a-5p	NAA25
miR-146a-5p	SIK1
miR-146a-5p	CXCL10
miR-146a-5p	PSMB9
miR-146a-5p	HFE
miR-146a-5p	TWIST1
miR-146a-5p	TNFRSF1B
miR-146a-5p	TNFAIP8
miR-146a-5p	FOSB
miR-146a-5p	SLC19A1
miR-146a-5p	PTPN2
miR-146a-5p	HLA-DPA1
miR-146a-5p	DUSP4
miR-146a-5p	HLA-C
miR-146a-5p	NGF
miR-146a-5p	JMJD1C
miR-146a-5p	PDCD1
miR-146a-5p	HLA-B
miR-146a-5p	GMPR
miR-146a-5p	TFDP1
miR-146a-5p	KSR1
miR-146a-5p	SIPA1L1
miR-146a-5p	AOPEP
miR-146a-5p	PRORP
miR-146a-5p	IRF1-AS1
miR-223-5p	FOS
miR-223-5p	SLC2A3
miR-223-5p	ERAP2
miR-223-5p	ATIC

Supplementary Table 5. List of genes (74) regulated by more than one of the identified miRNAs in patients uveitis (196 miRNA-gene interaction pairs).

miRNA	Gene
miR-125a-5p	MAPK14
miR-125a-5p	VEGFA
miR-125a-5p	CRK
miR-125a-5p	IFNG
miR-125a-5p	PIK3CG
miR-125a-5p	CCL5
miR-125a-5p	MAP2K7
miR-125a-5p	SOD2
miR-125a-5p	AMD1
miR-125a-5p	XIAP
miR-125a-5p	IL6ST
miR-125a-5p	NFE2L2
miR-125a-5p	SCAF11
miR-125a-5p	KIAA1109
miR-125a-5p	VCAN
miR-125a-5p	PTPN2
miR-140-5p	VEGFA
miR-140-5p	STAT1
miR-140-5p	ARIH1
miR-140-5p	ETS1
miR-140-5p	TCF4
miR-140-5p	RNF19A
miR-140-5p	POLDIP2
miR-140-5p	DPYSL5
miR-140-5p	EPRS1
miR-146a-5p	CXCR4
miR-146a-5p	CFH
miR-146a-5p	STAT1
miR-146a-5p	FAS
miR-146a-5p	TLR4
miR-146a-5p	ICAM1
miR-146a-5p	IL1RAP
miR-146a-5p	IL6
miR-146a-5p	CCL5
miR-146a-5p	ESD
miR-146a-5p	CXCL8
miR-146a-5p	VEGFA
miR-146a-5p	SOCS5
miR-146a-5p	SOCS3
miR-146a-5p	TGFB2
miR-146a-5p	SOCS1
miR-146a-5p	CD274
miR-146a-5p	RASGRP1
miR-146a-5p	KLF4
miR-146a-5p	SOD2
miR-146a-5p	ANXA11
miR-146a-5p	IL18
miR-146a-5p	PIK3CA
miR-146a-5p	PIK3CG
miR-146a-5p	PTPRF
miR-146a-5p	SMUG1
miR-146a-5p	ERAP1
miR-146a-5p	ERAP2
miR-146a-5p	TNFRSF1A
miR-146a-5p	VCAN
miR-146a-5p	ACAN
miR-146a-5p	PTPN2
miR-146a-5p	CKB
miR-146a-5p	HLA-C
miR-146a-5p	IFNB1
miR-146a-5p	BRAF
miR-146a-5p	HLA-B
miR-146a-5p	LCN2
miR-146a-5p	ARIH1
miR-146a-5p	RTN4R
miR-146a-5p	MAP2K7
miR-155-5p	ETS1
miR-155-5p	MYD88
miR-155-5p	SOCS1
miR-155-5p	PTEN
miR-155-5p	MAPK14
miR-155-5p	TJP1
miR-155-5p	SOCS3
miR-155-5p	SIRT1
miR-155-5p	ICAM1
miR-155-5p	STAT1
miR-155-5p	NR3C1
miR-155-5p	IL6
miR-155-5p	PIK3CA
miR-155-5p	TCF4
miR-155-5p	AIMP1
miR-155-5p	CXCL8
miR-155-5p	EPRS1

miR-155-5p	VEGFA		miR-223-3p	IL6ST
miR-155-5p	FAS		miR-223-3p	VEGFA
miR-155-5p	SOCS5		miR-223-3p	RASGRP1
miR-155-5p	TLR4		miR-223-5p	AMD1
miR-155-5p	TGFB2		miR-223-5p	IL6ST
miR-155-5p	RELA		miR-223-5p	AIMP1
miR-155-5p	CD274		miR-223-5p	TRAF5
miR-155-5p	RASGRP1		miR-223-5p	EGF
miR-155-5p	CXCR4		miR-223-5p	ETS1
miR-155-5p	KLF4		miR-223-5p	CRK
miR-155-5p	CFH		miR-223-5p	ERAP2
miR-155-5p	SOD2		miR-23a-3p	PTEN
miR-155-5p	ANXA11		miR-23a-3p	FAS
miR-155-5p	AMD1		miR-23a-3p	SOD2
miR-155-5p	XIAP		miR-23a-3p	XIAP
miR-155-5p	CRK		miR-23a-3p	CXCL8
miR-155-5p	IL1RAP		miR-23a-3p	TJP1
miR-155-5p	IL6ST		miR-23a-3p	AHR
miR-155-5p	IL18		miR-23a-3p	ANXA11
miR-155-5p	CD46		miR-23a-3p	NR3C1
miR-155-5p	NR3C2		miR-23a-3p	IFNG
miR-155-5p	NFE2L2		miR-23a-3p	IL6ST
miR-155-5p	PIK3CB		miR-23a-3p	CD46
miR-155-5p	MAPK1		miR-23a-3p	MYD88
miR-155-5p	PTPRF		miR-23a-3p	NFE2L2
miR-155-5p	VIP		miR-23a-3p	PIK3CB
miR-155-5p	SMUG1		miR-23a-3p	MAPK1
miR-155-5p	RNF19A		miR-23a-3p	VIP
miR-155-5p	ATN1		miR-23a-3p	RNF19A
miR-155-5p	VCAN		miR-23a-3p	KIAA1109
miR-155-5p	EGF		miR-23a-3p	ATN1
miR-155-5p	AQP1		miR-23a-3p	TRAF5
miR-155-5p	ESD		miR-23a-3p	VCAN
miR-155-5p	HLA-C		miR-23a-3p	HLA-C
miR-155-5p	BRAF		miR-23a-3p	IFNB1
miR-155-5p	HLA-B		miR-23a-3p	HLA-B
miR-155-5p	LCN2		miR-23a-3p	ARIH1
miR-155-5p	ARIH1		miR-23a-3p	ATF7IP
miR-155-5p	ATF7IP		miR-29a-3p	PTEN
miR-155-5p	RTN4R		miR-29a-3p	VEGFA
miR-193a-5p	ANXA11		miR-29a-3p	AHR
miR-193a-5p	ERAP1		miR-29a-3p	RASGRP1
miR-193a-5p	ACAN		miR-29a-3p	KLF4
miR-193a-5p	EPRS1		miR-29a-3p	TJP1
miR-223-3p	STAT1		miR-29a-3p	TGFB2
miR-223-3p	IL6		miR-29a-3p	RELA

miR-29a-3p	SIRT1
miR-29a-3p	NR3C1
miR-29a-3p	CD46
miR-29a-3p	NR3C2
miR-29a-3p	NFE2L2
miR-29a-3p	PTPRF
miR-29a-3p	TCF4
miR-29a-3p	SCAF11
miR-29a-3p	RNF19A
miR-29a-3p	POLDIP2
miR-29a-3p	ERAP1

miR-29a-3p	DPYSL5
miR-29a-3p	TRAF5
miR-29a-3p	TNFRSF1A
miR-29a-3p	VCAN
miR-29a-3p	AQP1
miR-29a-3p	CKB
miR-29a-3p	CXCL8
miR-29a-3p	EPRS1
miR-491-5p	HLA-C
miR-491-5p	ATN1

Supplementary Table 6. Experimentally validated miRNA:gene pairs found *in silico* among 9 the most frequently detected miRNAs in patients with juvenile arthritis-associated uveitis and 450 genes associated with juvenile arthritis (648 miRNA-gene interaction pairs). From this group, 299 unique genes were selected for pathway enrichment analysis.

**miRNA (JIA-AU)**	**Target gene**
hsa-miR-16-5p	BCL2
hsa-miR-16-5p	VEGFA
hsa-miR-16-5p	CARD8
hsa-miR-16-5p	JUN
hsa-miR-16-5p	CDK6
hsa-miR-155-5p	MeCP2
hsa-miR-125a-5p	TP53
hsa-miR-146a-5p	IRAK1
hsa-miR-146a-5p	CXCR4
hsa-miR-146a-5p	STAT1
hsa-miR-451a	ABCB1
hsa-miR-451a	MIF
hsa-miR-26a-5p	CDK6
hsa-miR-26a-5p	HSPD1
hsa-miR-26a-5p	ESR1
hsa-miR-26a-5p	ATM
hsa-miR-26a-5p	RHOBTB1
hsa-miR-26a-5p	SLC26A2
hsa-miR-26a-5p	IL6
hsa-miR-26a-5p	PLOD2
hsa-miR-26a-5p	NAMPT
hsa-miR-26a-5p	FAM177A1
hsa-miR-26a-5p	IGF1
hsa-miR-26a-5p	DUSP4
hsa-miR-26a-5p	PSMA3
hsa-miR-26a-5p	TTN
hsa-miR-145-5p	CDK6
hsa-miR-145-5p	ESR1
hsa-miR-145-5p	VEGFA
hsa-miR-145-5p	HIF1A
hsa-miR-145-5p	KLF4
hsa-miR-145-5p	MMP1
hsa-miR-145-5p	STAT1
hsa-miR-145-5p	BTG1
hsa-miR-145-5p	CD28
hsa-miR-145-5p	SLC26A2
hsa-miR-145-5p	HBEGF
hsa-miR-145-5p	IGF1R
hsa-miR-145-5p	SMAD3
hsa-miR-145-5p	DEK
hsa-miR-145-5p	ZFAND3
hsa-miR-16-5p	HSPD1
hsa-miR-16-5p	KRAS
hsa-miR-16-5p	CLU
hsa-miR-16-5p	VIM
hsa-miR-16-5p	CD274
hsa-miR-16-5p	TP53
hsa-miR-16-5p	ENTPD1
hsa-miR-16-5p	HMOX1
hsa-miR-16-5p	IFNG
hsa-miR-16-5p	IGF1R
hsa-miR-16-5p	KIF5A
hsa-miR-16-5p	LIG4
hsa-miR-16-5p	SMAD3
hsa-miR-16-5p	MTHFR
hsa-miR-16-5p	PLAUR
hsa-miR-16-5p	PRKAR1A
hsa-miR-16-5p	PRNP
hsa-miR-16-5p	SLC2A3
hsa-miR-16-5p	TFPI
hsa-miR-16-5p	UBE3C
hsa-miR-16-5p	BMS1
hsa-miR-16-5p	NAMPT
hsa-miR-16-5p	ATXN2L
hsa-miR-16-5p	FNBP1
hsa-miR-16-5p	AVL9
hsa-miR-16-5p	TTLL5
hsa-miR-16-5p	ETNK1
hsa-miR-16-5p	NAA25
hsa-miR-16-5p	SIK1
hsa-miR-16-5p	CAMSAP1
hsa-miR-16-5p	LURAP1L
hsa-miR-16-5p	TUBB2A
hsa-miR-16-5p	SLC19A1
hsa-miR-16-5p	PDCD1
hsa-miR-223-3p	ABCB1
hsa-miR-223-3p	TP53
hsa-miR-223-3p	STAT1
hsa-miR-223-3p	ATM
hsa-miR-223-3p	STAT3
hsa-miR-223-3p	IGF1R
hsa-miR-223-3p	IL6

hsa-miR-223-3p	IL6ST
hsa-miR-223-3p	NAMPT
hsa-miR-223-3p	NLRP3
hsa-miR-223-3p	CCL3
hsa-miR-155-5p	THBS1
hsa-miR-155-5p	KRAS
hsa-miR-155-5p	VCAM1
hsa-miR-155-5p	JUN
hsa-miR-155-5p	HIF1A
hsa-miR-155-5p	FOS
hsa-miR-155-5p	ICAM1
hsa-miR-155-5p	STAT1
hsa-miR-155-5p	STAT3
hsa-miR-155-5p	CAT
hsa-miR-155-5p	ENTPD1
hsa-miR-155-5p	CTLA4
hsa-miR-155-5p	CTNNB1
hsa-miR-155-5p	DAG1
hsa-miR-155-5p	FLNB
hsa-miR-155-5p	IL2
hsa-miR-155-5p	IL6
hsa-miR-155-5p	ITGB5
hsa-miR-155-5p	SMAD3
hsa-miR-155-5p	NOS3
hsa-miR-155-5p	NT5E
hsa-miR-155-5p	OLR1
hsa-miR-155-5p	PLAUR
hsa-miR-155-5p	PRKAR1A
hsa-miR-155-5p	CCL2
hsa-miR-155-5p	SELE
hsa-miR-155-5p	STIM1
hsa-miR-155-5p	STXBP2
hsa-miR-155-5p	TFPI
hsa-miR-155-5p	TGM2
hsa-miR-155-5p	DEK
hsa-miR-155-5p	NAMPT
hsa-miR-155-5p	PACSIN2
hsa-miR-155-5p	MEX3C
hsa-miR-155-5p	NAA25
hsa-miR-155-5p	FAM177A1
hsa-miR-155-5p	HLA-DPA1
hsa-miR-155-5p	CXCL8
hsa-miR-125a-5p	BCL2
hsa-miR-125a-5p	VEGFA
hsa-miR-125a-5p	IL1RN
hsa-miR-125a-5p	STAT3

hsa-miR-125a-5p	IFNG
hsa-miR-125a-5p	CCL5
hsa-miR-125a-5p	TNFAIP3
hsa-miR-125a-5p	NDEL1
hsa-miR-146a-5p	FAS
hsa-miR-146a-5p	TLR4
hsa-miR-146a-5p	TGFB1
hsa-miR-146a-5p	FOS
hsa-miR-146a-5p	ICAM1
hsa-miR-146a-5p	TLR2
hsa-miR-146a-5p	MIF
hsa-miR-146a-5p	BGLAP
hsa-miR-146a-5p	DUSP1
hsa-miR-146a-5p	IL6
hsa-miR-146a-5p	PLAUR
hsa-miR-146a-5p	CCL5
hsa-miR-146a-5p	SPP1
hsa-miR-146a-5p	AVL9
hsa-miR-146a-5p	S100A12
hsa-miR-146a-5p	CXCL8
hsa-miR-451a	BCL2
hsa-miR-451a	IL6R
hsa-miR-451a	MMP9
hsa-miR-451a	IL6
hsa-miR-451a	MMP2
hsa-miR-451a	MAPK1
hsa-miR-223-5p	ETV6
hsa-miR-223-5p	FOXP1
hsa-miR-223-5p	AR
hsa-miR-223-5p	IGF1R
hsa-miR-223-5p	IL6ST
hsa-miR-223-5p	LPP
hsa-miR-223-5p	TRIM13
hsa-miR-223-5p	CD226
hsa-miR-223-5p	NAA25
hsa-miR-223-5p	FAM177A1
hsa-miR-223-5p	IGF1
hsa-miR-223-5p	TNFAIP8
hsa-miR-223-5p	EGF
hsa-miR-26a-5p	VCAM1
hsa-miR-26a-5p	FBN1
hsa-miR-26a-5p	JUN
hsa-miR-26a-5p	VIM
hsa-miR-26a-5p	FOS
hsa-miR-26a-5p	STAT1
hsa-miR-26a-5p	STAT3

hsa-miR-26a-5p	BTG1
hsa-miR-26a-5p	C5
hsa-miR-26a-5p	CALD1
hsa-miR-26a-5p	CAT
hsa-miR-26a-5p	CCR4
hsa-miR-26a-5p	CCR7
hsa-miR-26a-5p	CXADR
hsa-miR-26a-5p	CTTN
hsa-miR-26a-5p	GSTP1
hsa-miR-26a-5p	HLA-A
hsa-miR-26a-5p	HLA-DQA1
hsa-miR-26a-5p	HLA-G
hsa-miR-26a-5p	IGF1R
hsa-miR-26a-5p	IL6ST
hsa-miR-26a-5p	CXCR2
hsa-miR-26a-5p	IRF1
hsa-miR-26a-5p	MAP2
hsa-miR-26a-5p	NOS3
hsa-miR-26a-5p	PLAUR
hsa-miR-26a-5p	PTX3
hsa-miR-26a-5p	SLC2A3
hsa-miR-26a-5p	SNAI1
hsa-miR-26a-5p	TIMP1
hsa-miR-26a-5p	TIMP2
hsa-miR-26a-5p	UBE3C
hsa-miR-26a-5p	AVL9
hsa-miR-26a-5p	ASAP1
hsa-miR-26a-5p	MEX3C
hsa-miR-26a-5p	ETNK1
hsa-miR-26a-5p	ZMIZ1
hsa-miR-26a-5p	NAA25
hsa-miR-26a-5p	NDEL1
hsa-miR-26a-5p	SIK1
hsa-miR-26a-5p	CAMSAP1
hsa-miR-26a-5p	ZFC3H1
hsa-miR-26a-5p	REEP3
hsa-miR-26a-5p	BRD2
hsa-miR-26a-5p	TWIST1
hsa-miR-26a-5p	LTA
hsa-miR-26a-5p	TLR10
hsa-miR-26a-5p	TUBB2A
hsa-miR-26a-5p	FOSB
hsa-miR-26a-5p	TCF7L2
hsa-miR-26a-5p	CD8B
hsa-miR-26a-5p	ATG16L1
hsa-miR-26a-5p	RNF103
hsa-miR-26a-5p	PRKCQ
hsa-miR-26a-5p	HLA-DPA1
hsa-miR-26a-5p	HLA-DQB1
hsa-miR-26a-5p	GNG11
hsa-miR-26a-5p	JMJD1C
hsa-miR-26a-5p	FOSL2
hsa-miR-26a-5p	HLA-B
hsa-miR-26a-5p	SIPA1L1
hsa-miR-26a-5p	HYAL1
hsa-miR-26a-5p	BEND2
hsa-miR-26a-5p	PSTPIP1
hsa-miR-26a-5p	HLA-DPB1
hsa-miR-26a-5p	MIF
hsa-miR-26a-5p	PRORP
hsa-miR-145-5p	THBS1
hsa-miR-145-5p	IL6R
hsa-miR-145-5p	FBN1
hsa-miR-145-5p	JUN
hsa-miR-145-5p	IL1RN
hsa-miR-145-5p	VIM
hsa-miR-145-5p	TGFB1
hsa-miR-145-5p	MMP3
hsa-miR-145-5p	MMP9
hsa-miR-145-5p	ATM
hsa-miR-145-5p	STAT3
hsa-miR-145-5p	CALD1
hsa-miR-145-5p	CTNNB1
hsa-miR-145-5p	MMP2
hsa-miR-145-5p	MSN
hsa-miR-145-5p	PRNP
hsa-miR-145-5p	CCL20
hsa-miR-145-5p	SNAI1
hsa-miR-145-5p	SPP1
hsa-miR-145-5p	TIMP1
hsa-miR-145-5p	TNFAIP3
hsa-miR-145-5p	SH2B3
hsa-miR-145-5p	NAMPT
hsa-miR-145-5p	ETNK1
hsa-miR-145-5p	TWIST1
hsa-miR-145-5p	POSTN
hsa-miR-145-5p	GNG11
hsa-miR-145-5p	JMJD1C
hsa-miR-145-5p	SIPA1L1
hsa-miR-16-5p	THBS1
hsa-miR-16-5p	MTX1
hsa-miR-16-5p	FAS

hsa-miR-16-5p	IL6R
hsa-miR-16-5p	RERE
hsa-miR-16-5p	FBN1
hsa-miR-16-5p	TLR4
hsa-miR-16-5p	HSPA14
hsa-miR-16-5p	CD83
hsa-miR-16-5p	ABCB1
hsa-miR-16-5p	HIF1A
hsa-miR-16-5p	TGFB1
hsa-miR-16-5p	CXCR4
hsa-miR-16-5p	MMP3
hsa-miR-16-5p	ELK3
hsa-miR-16-5p	FOS
hsa-miR-16-5p	NR4A2
hsa-miR-16-5p	ICAM1
hsa-miR-16-5p	KLF4
hsa-miR-16-5p	ETV6
hsa-miR-16-5p	STAT1
hsa-miR-16-5p	DUSP2
hsa-miR-16-5p	NCAM1
hsa-miR-16-5p	ANXA11
hsa-miR-16-5p	RHOBTB1
hsa-miR-16-5p	STAT3
hsa-miR-16-5p	ADA
hsa-miR-16-5p	AR
hsa-miR-16-5p	AREG
hsa-miR-16-5p	BTG1
hsa-miR-16-5p	CALD1
hsa-miR-16-5p	CAT
hsa-miR-16-5p	CTNNB1
hsa-miR-16-5p	DAG1
hsa-miR-16-5p	DAPK1
hsa-miR-16-5p	DDIT3
hsa-miR-16-5p	DHCR7
hsa-miR-16-5p	DUSP1
hsa-miR-16-5p	EGR1
hsa-miR-16-5p	FLNB
hsa-miR-16-5p	GATA3
hsa-miR-16-5p	GEM
hsa-miR-16-5p	CXCL1
hsa-miR-16-5p	GSTP1
hsa-miR-16-5p	HLA-A
hsa-miR-16-5p	IL1A
hsa-miR-16-5p	IL6
hsa-miR-16-5p	IL6ST
hsa-miR-16-5p	IL7R

hsa-miR-16-5p	IRF1
hsa-miR-16-5p	ISG20
hsa-miR-16-5p	ITGB5
hsa-miR-16-5p	LPP
hsa-miR-16-5p	MAP2
hsa-miR-16-5p	MME
hsa-miR-16-5p	MSN
hsa-miR-16-5p	NT5E
hsa-miR-16-5p	TNFRSF11B
hsa-miR-16-5p	PER1
hsa-miR-16-5p	PLOD2
hsa-miR-16-5p	MAPK1
hsa-miR-16-5p	PROS1
hsa-miR-16-5p	PSMC6
hsa-miR-16-5p	PTX3
hsa-miR-16-5p	CCL2
hsa-miR-16-5p	SPP1
hsa-miR-16-5p	TGM2
hsa-miR-16-5p	TIMP2
hsa-miR-16-5p	TNFAIP3
hsa-miR-16-5p	UBE2E1
hsa-miR-16-5p	NR4A3
hsa-miR-16-5p	USO1
hsa-miR-16-5p	ABCC3
hsa-miR-16-5p	IL32
hsa-miR-16-5p	B4GALT5
hsa-miR-16-5p	MTSS1
hsa-miR-16-5p	TNFSF15
hsa-miR-16-5p	SH2B3
hsa-miR-16-5p	TRIM13
hsa-miR-16-5p	PLK2
hsa-miR-16-5p	PACSIN2
hsa-miR-16-5p	NLRP1
hsa-miR-16-5p	TNIK
hsa-miR-16-5p	CRB1
hsa-miR-16-5p	MAFF
hsa-miR-16-5p	NRBF2
hsa-miR-16-5p	ASAP1
hsa-miR-16-5p	RSBN1
hsa-miR-16-5p	CXCL16
hsa-miR-16-5p	VTCN1
hsa-miR-16-5p	EBPL
hsa-miR-16-5p	NKD1
hsa-miR-16-5p	ANKRD9
hsa-miR-16-5p	ZFC3H1
hsa-miR-16-5p	REEP3

hsa-miR-16-5p	BRD2
hsa-miR-16-5p	CXCL10
hsa-miR-16-5p	IGF1
hsa-miR-16-5p	PSMB9
hsa-miR-16-5p	ADRB2
hsa-miR-16-5p	HFE
hsa-miR-16-5p	IL4R
hsa-miR-16-5p	CCR5
hsa-miR-16-5p	TAP1
hsa-miR-16-5p	IL2RB
hsa-miR-16-5p	ATN1
hsa-miR-16-5p	TNFAIP8
hsa-miR-16-5p	FOSB
hsa-miR-16-5p	DYSF
hsa-miR-16-5p	TCF7L2
hsa-miR-16-5p	LRBA
hsa-miR-16-5p	BMP4
hsa-miR-16-5p	DUSP4
hsa-miR-16-5p	HSPA6
hsa-miR-16-5p	NGF
hsa-miR-16-5p	ATIC
hsa-miR-16-5p	GNG11
hsa-miR-16-5p	JMJD1C
hsa-miR-16-5p	FOSL2
hsa-miR-16-5p	GMPR
hsa-miR-16-5p	TFDP1
hsa-miR-16-5p	TNFAIP6
hsa-miR-16-5p	ACAD8
hsa-miR-16-5p	SIPA1L1
hsa-miR-16-5p	WWOX
hsa-miR-16-5p	SH3BGRL2
hsa-miR-16-5p	HYAL1
hsa-miR-16-5p	CTTNBP2
hsa-miR-16-5p	ITPA
hsa-miR-16-5p	RASGEF1B
hsa-miR-16-5p	PSTPIP1
hsa-miR-16-5p	CXCL8
hsa-miR-16-5p	GSTT1
hsa-miR-16-5p	ADGRL2
hsa-miR-16-5p	ACACA
hsa-miR-16-5p	AOPEP
hsa-miR-16-5p	PRORP
hsa-miR-16-5p	H2BC8
hsa-miR-16-5p	IRF1-AS1
hsa-miR-223-3p	BCL2
hsa-miR-223-3p	VEGFA

hsa-miR-223-3p	MMP9
hsa-miR-223-3p	CAT
hsa-miR-223-3p	ENTPD1
hsa-miR-223-3p	MECP2
hsa-miR-223-3p	MMP2
hsa-miR-155-5p	BCL2
hsa-miR-155-5p	CDK6
hsa-miR-155-5p	HSPD1
hsa-miR-155-5p	MTX1
hsa-miR-155-5p	RBM47
hsa-miR-155-5p	VEGFA
hsa-miR-155-5p	FAS
hsa-miR-155-5p	IL6R
hsa-miR-155-5p	RERE
hsa-miR-155-5p	FBN1
hsa-miR-155-5p	TLR4
hsa-miR-155-5p	CD83
hsa-miR-155-5p	CLU
hsa-miR-155-5p	ABCB1
hsa-miR-155-5p	VIM
hsa-miR-155-5p	CD274
hsa-miR-155-5p	CXCR4
hsa-miR-155-5p	TP53
hsa-miR-155-5p	ELK3
hsa-miR-155-5p	NR4A2
hsa-miR-155-5p	KLF4
hsa-miR-155-5p	MMP1
hsa-miR-155-5p	ANXA11
hsa-miR-155-5p	RHOBTB1
hsa-miR-155-5p	HERPUD2
hsa-miR-155-5p	ADA
hsa-miR-155-5p	CALD1
hsa-miR-155-5p	DAPK1
hsa-miR-155-5p	GADD45A
hsa-miR-155-5p	DDIT3
hsa-miR-155-5p	SLC26A2
hsa-miR-155-5p	DUSP1
hsa-miR-155-5p	EGR1
hsa-miR-155-5p	CTTN
hsa-miR-155-5p	GAS6
hsa-miR-155-5p	GEM
hsa-miR-155-5p	CXCL1
hsa-miR-155-5p	HMOX1
hsa-miR-155-5p	IL1B
hsa-miR-155-5p	IL6ST
hsa-miR-155-5p	IL7R

hsa-miR-155-5p	IL10
hsa-miR-155-5p	IL18
hsa-miR-155-5p	ISG20
hsa-miR-155-5p	LIG4
hsa-miR-155-5p	LPP
hsa-miR-155-5p	NFKBIA
hsa-miR-155-5p	TNFRSF11B
hsa-miR-155-5p	PLOD2
hsa-miR-155-5p	MAPK1
hsa-miR-155-5p	PRNP
hsa-miR-155-5p	PROS1
hsa-miR-155-5p	PTX3
hsa-miR-155-5p	RAB27A
hsa-miR-155-5p	SLC2A3
hsa-miR-155-5p	TIMP2
hsa-miR-155-5p	TNF
hsa-miR-155-5p	TNFAIP3
hsa-miR-155-5p	UBE2E1
hsa-miR-155-5p	USO1
hsa-miR-155-5p	ABCC3
hsa-miR-155-5p	IL32
hsa-miR-155-5p	B4GALT5
hsa-miR-155-5p	BMS1
hsa-miR-155-5p	FCHSD2
hsa-miR-155-5p	SH2B3
hsa-miR-155-5p	PLK2
hsa-miR-155-5p	ATXN2L
hsa-miR-155-5p	TNIK
hsa-miR-155-5p	CRB1
hsa-miR-155-5p	MAFF
hsa-miR-155-5p	ASAP1
hsa-miR-155-5p	ETNK1
hsa-miR-155-5p	MAML2
hsa-miR-155-5p	ANKRD9
hsa-miR-155-5p	LURAP1L
hsa-miR-155-5p	BRD2
hsa-miR-155-5p	IGF1
hsa-miR-155-5p	ADRB2
hsa-miR-155-5p	IL4R
hsa-miR-155-5p	LTA
hsa-miR-155-5p	IL2RB
hsa-miR-155-5p	ATN1
hsa-miR-155-5p	DCLRE1C
hsa-miR-155-5p	TNFAIP8
hsa-miR-155-5p	POSTN
hsa-miR-155-5p	TCF7L2

hsa-miR-155-5p	EGF
hsa-miR-155-5p	ATG16L1
hsa-miR-155-5p	LRBA
hsa-miR-155-5p	SIAE
hsa-miR-155-5p	BMP4
hsa-miR-155-5p	HLA-C
hsa-miR-155-5p	HSPA6
hsa-miR-155-5p	PTPRC
hsa-miR-155-5p	GNG11
hsa-miR-155-5p	FOSL2
hsa-miR-155-5p	HLA-B
hsa-miR-155-5p	GMPR
hsa-miR-155-5p	TFDP1
hsa-miR-155-5p	TNFAIP6
hsa-miR-155-5p	KSR1
hsa-miR-155-5p	WWOX
hsa-miR-155-5p	IL23R
hsa-miR-155-5p	ADGRL2
hsa-miR-155-5p	ACACA
hsa-miR-155-5p	AOPEP
hsa-miR-125a-5p	THBS1
hsa-miR-125a-5p	JUN
hsa-miR-125a-5p	IRAK1
hsa-miR-125a-5p	NCAM1
hsa-miR-125a-5p	CTNNB1
hsa-miR-125a-5p	IL6ST
hsa-miR-125a-5p	PRKAR1A
hsa-miR-125a-5p	UBE3C
hsa-miR-125a-5p	TNFSF15
hsa-miR-125a-5p	ATXN2L
hsa-miR-125a-5p	ZNF395
hsa-miR-125a-5p	ZFC3H1
hsa-miR-125a-5p	TNFRSF1B
hsa-miR-125a-5p	PTPN2
hsa-miR-125a-5p	DUSP4
hsa-miR-125a-5p	ELF2
hsa-miR-125a-5p	SIPA1L1
hsa-miR-146a-5p	CDK6
hsa-miR-146a-5p	THBS1
hsa-miR-146a-5p	VEGFA
hsa-miR-146a-5p	RERE
hsa-miR-146a-5p	CD83
hsa-miR-146a-5p	CARD8
hsa-miR-146a-5p	JUN
hsa-miR-146a-5p	CLU
hsa-miR-146a-5p	CD274

hsa-miR-146a-5p	MMP9
hsa-miR-146a-5p	KLF4
hsa-miR-146a-5p	FOXP1
hsa-miR-146a-5p	ANXA11
hsa-miR-146a-5p	CALD1
hsa-miR-146a-5p	CD247
hsa-miR-146a-5p	CD28
hsa-miR-146a-5p	CCR4
hsa-miR-146a-5p	CXADR
hsa-miR-146a-5p	SLC26A2
hsa-miR-146a-5p	FLNB
hsa-miR-146a-5p	GAS6
hsa-miR-146a-5p	CXCL1
hsa-miR-146a-5p	HLA-G
hsa-miR-146a-5p	IL18
hsa-miR-146a-5p	ITGB5
hsa-miR-146a-5p	LPP
hsa-miR-146a-5p	MECP2
hsa-miR-146a-5p	CIITA
hsa-miR-146a-5p	MME
hsa-miR-146a-5p	NOS3
hsa-miR-146a-5p	PGM5
hsa-miR-146a-5p	SAG
hsa-miR-146a-5p	CCL19
hsa-miR-146a-5p	CCL20
hsa-miR-146a-5p	SLC2A3
hsa-miR-146a-5p	SNAI1
hsa-miR-146a-5p	STIM1
hsa-miR-146a-5p	STXBP2
hsa-miR-146a-5p	TIMP1
hsa-miR-146a-5p	TIMP2
hsa-miR-146a-5p	TNFAIP3
hsa-miR-146a-5p	USO1
hsa-miR-146a-5p	ABCC3
hsa-miR-146a-5p	IL32
hsa-miR-146a-5p	SH2B3
hsa-miR-146a-5p	TRIM13
hsa-miR-146a-5p	CCL27
hsa-miR-146a-5p	TNIK
hsa-miR-146a-5p	FNBP1
hsa-miR-146a-5p	TTLL5
hsa-miR-146a-5p	PTPN22
hsa-miR-146a-5p	ASAP1
hsa-miR-146a-5p	MEX3C
hsa-miR-146a-5p	ERAP1
hsa-miR-146a-5p	ETNK1

hsa-miR-146a-5p	CXCL16
hsa-miR-146a-5p	ERAP2
hsa-miR-146a-5p	NAA25
hsa-miR-146a-5p	NT5C1A
hsa-miR-146a-5p	IL33
hsa-miR-146a-5p	SIK1
hsa-miR-146a-5p	UNC13D
hsa-miR-146a-5p	LILRA5
hsa-miR-146a-5p	CXCL10
hsa-miR-146a-5p	PSMB9
hsa-miR-146a-5p	COMP
hsa-miR-146a-5p	HFE
hsa-miR-146a-5p	ITGA2B
hsa-miR-146a-5p	TWIST1
hsa-miR-146a-5p	FUT2
hsa-miR-146a-5p	C4BPA
hsa-miR-146a-5p	CSF3
hsa-miR-146a-5p	CACNA1I
hsa-miR-146a-5p	TNFRSF1A
hsa-miR-146a-5p	TNFRSF1B
hsa-miR-146a-5p	NR1I3
hsa-miR-146a-5p	TNFAIP8
hsa-miR-146a-5p	ACP5
hsa-miR-146a-5p	FOSB
hsa-miR-146a-5p	ACAN
hsa-miR-146a-5p	CXCR3
hsa-miR-146a-5p	CALCR
hsa-miR-146a-5p	GPR35
hsa-miR-146a-5p	NCR2
hsa-miR-146a-5p	SEC14L2
hsa-miR-146a-5p	SLC19A1
hsa-miR-146a-5p	PTPN2
hsa-miR-146a-5p	HLA-DPA1
hsa-miR-146a-5p	DUSP4
hsa-miR-146a-5p	CREM
hsa-miR-146a-5p	DPP4
hsa-miR-146a-5p	HLA-C
hsa-miR-146a-5p	NGF
hsa-miR-146a-5p	SELP
hsa-miR-146a-5p	TNFRSF10C
hsa-miR-146a-5p	GZMB
hsa-miR-146a-5p	JMJD1C
hsa-miR-146a-5p	PDCD1
hsa-miR-146a-5p	HLA-B
hsa-miR-146a-5p	GMPR
hsa-miR-146a-5p	TFDP1

hsa-miR-146a-5p	KSR1
hsa-miR-146a-5p	SIPA1L1
hsa-miR-146a-5p	OSM
hsa-miR-146a-5p	PYDC1
hsa-miR-146a-5p	ACRBP
hsa-miR-146a-5p	CRYGD
hsa-miR-146a-5p	TREML1

hsa-miR-146a-5p	AOPEP
hsa-miR-146a-5p	PRORP
hsa-miR-146a-5p	IRF1-AS1
hsa-miR-223-5p	FOS
hsa-miR-223-5p	SLC2A3
hsa-miR-223-5p	ERAP2
hsa-miR-223-5p	ATIC

**Genes (299)**	VIM	DAG1	TNFAIP8	HLA-B
BCL2	CD274	FLNB	EGF	SIPA1L1
VEGFA	ENTPD1	IL2	FBN1	HYAL1
CARD8	HMOX1	ITGB5	C5	BEND2
JUN	IFNG	NOS3	CALD1	PSTPIP1
CDK6	KIF5A	NT5E	CCR4	HLA-DPB1
MeCP2	LIG4	OLR1	CCR7	PRORP
TP53	MTHFR	CCL2	CXADR	MMP3
IRAK1	PLAUR	SELE	CTTN	MSN
CXCR4	PRKAR1A	STIM1	GSTP1	CCL20
STAT1	PRNP	STXBP2	HLA-A	SH2B3
ABCB1	SLC2A3	TGM2	HLA-DQA1	POSTN
MIF	TFPI	PACSIN2	HLA-G	MTX1
HSPD1	UBE3C	MEX3C	CXCR2	RERE
ESR1	BMS1	HLA-DPA1	IRF1	HSPA14
ATM	ATXN2L	CXCL8	MAP2	CD83
RHOBTB1	FNBP1	IL1RN	PTX3	ELK3
SLC26A2	AVL9	CCL5	SNAI1	NR4A2
IL6	TTLL5	TNFAIP3	TIMP1	DUSP2
PLOD2	ETNK1	NDEL1	TIMP2	NCAM1
NAMPT	NAA25	FAS	ASAP1	ANXA11
FAM177A1	SIK1	TLR4	ZMIZ1	ADA
IGF1	CAMSAP1	TGFB1	ZFC3H1	AREG
DUSP4	LURAP1L	TLR2	REEP3	DAPK1
PSMA3	TUBB2A	BGLAP	BRD2	DDIT3
TTN	SLC19A1	DUSP1	TWIST1	DHCR7
HIF1A	PDCD1	SPP1	LTA	EGR1
KLF4	STAT3	S100A12	TLR10	GATA3
MMP1	IL6ST	IL6R	FOSB	GEM
BTG1	NLRP3	MMP9	TCF7L2	CXCL1
CD28	CCL3	MMP2	CD8B	IL1A
HBEGF	THBS1	MAPK1	ATG16L1	IL7R
IGF1R	VCAM1	ETV6	RNF103	ISG20
SMAD3	FOS	FOXP1	PRKCQ	MME
DEK	ICAM1	AR	HLA-DQB1	TNFRSF11B
ZFAND3	CAT	LPP	GNG11	PER1
KRAS	CTLA4	TRIM13	JMJD1C	PROS1
CLU	CTNNB1	CD226	FOSL2	PSMC6

UBE2E1	ADRB2	GSTT1	KSR1	C4BPA
NR4A3	HFE	ADGRL2	IL23R	CSF3
USO1	IL4R	ACACA	ZNF395	CACNA1I
ABCC3	CCR5	AOPEP	TNFRSF1B	TNFRSF1A
IL32	TAP1	H2BC8	PTPN2	NR1I3
B4GALT5	IL2RB	IRF1-AS1	ELF2	ACP5
MTSS1	ATN1	RBM47	CD247	ACAN
TNFSF15	DYSF	HERPUD2	CIITA	CXCR3
PLK2	LRBA	GADD45A	PGM5	CALCR
NLRP1	BMP4	GAS6	SAG	GPR35
TNIK	HSPA6	IL1B	CCL19	NCR2
CRB1	NGF	IL10	CCL27	SEC14L2
MAFF	ATIC	IL18	PTPN22	CREM
NRBF2	GMPR	NFKBIA	ERAP1	DPP4
RSBN1	TFDP1	RAB27A	ERAP2	SELP
CXCL16	TNFAIP6	TNF	NT5C1A	TNFRSF10C
VTCN1	ACAD8	FCHSD2	IL33	GZMB
EBPL	WWOX	MAML2	UNC13D	OSM
NKD1	SH3BGRL2	DCLRE1C	LILRA5	PYDC1
ANKRD9	CTTNBP2	SIAE	COMP	ACRBP
CXCL10	ITPA	HLA-C	ITGA2B	CRYGD
PSMB9	RASGEF1B	PTPRC	FUT2	TREML1

Supplementary Table 7. Experimentally validated miRNA:gene pairs found *in silico* among 10 the most frequently detected miRNAs in patients with uveitis and 247 genes associated with uveitis (264 miRNA-gene interaction pairs). From this group, 142 unique genes were selected for pathway enrichment analysis.

miRNA (IU)	Target gene
hsa-miR-155-5p	ETS1
hsa-miR-155-5p	MyD88
hsa-miR-155-5p	SOCS1
hsa-miR-140-5p	VEGFA
hsa-miR-29a-3p	PTEN
hsa-miR-146a-5p	CXCR4
hsa-miR-146a-5p	CFH
hsa-miR-146a-5p	STAT1
hsa-miR-23a-3p	PTEN
hsa-miR-23a-3p	FAS
hsa-miR-23a-3p	SOD2
hsa-miR-23a-3p	XIAP
hsa-miR-23a-3p	CCL8
hsa-miR-23a-3p	CXCL8
hsa-miR-223-3p	STAT1
hsa-miR-223-3p	IL6
hsa-miR-223-3p	IL6ST
hsa-miR-223-3p	NLRP3
hsa-miR-223-3p	TRPV2
hsa-miR-155-5p	PTEN
hsa-miR-155-5p	MAPK14
hsa-miR-155-5p	TJP1
hsa-miR-155-5p	SOCS3
hsa-miR-155-5p	SIRT1
hsa-miR-155-5p	ICAM1
hsa-miR-155-5p	STAT1
hsa-miR-155-5p	CTLA4
hsa-miR-155-5p	NR3C1
hsa-miR-155-5p	IL2
hsa-miR-155-5p	IL6
hsa-miR-155-5p	PIK3CA
hsa-miR-155-5p	CCL2
hsa-miR-155-5p	TCF4
hsa-miR-155-5p	AIMP1
hsa-miR-155-5p	SLC9A3R2
hsa-miR-155-5p	CXCL8
hsa-miR-155-5p	EPRS1
hsa-miR-125a-5p	MAPK14
hsa-miR-125a-5p	ZEB2
hsa-miR-125a-5p	VEGFA
hsa-miR-125a-5p	IL1RN
hsa-miR-125a-5p	CRK
hsa-miR-125a-5p	IFNG
hsa-miR-125a-5p	PIK3CG
hsa-miR-125a-5p	CCL5
hsa-miR-125a-5p	MAP2K7
hsa-miR-140-5p	STAT1
hsa-miR-140-5p	ARIH1
hsa-miR-29a-3p	VEGFA
hsa-miR-29a-3p	AHR
hsa-miR-29a-3p	RASGRP1
hsa-miR-29a-3p	KLF4
hsa-miR-29a-3p	S100B
hsa-miR-146a-5p	FAS
hsa-miR-146a-5p	TLR4
hsa-miR-146a-5p	ICAM1
hsa-miR-146a-5p	TLR2
hsa-miR-146a-5p	IL1RAP
hsa-miR-146a-5p	IL6
hsa-miR-146a-5p	CCL5
hsa-miR-146a-5p	DECR1
hsa-miR-146a-5p	ESD
hsa-miR-146a-5p	GRAP2
hsa-miR-146a-5p	S100A12
hsa-miR-146a-5p	CXCL8
hsa-miR-491-5p	HLA-C
hsa-miR-223-5p	AMD1
hsa-miR-223-5p	IL6ST
hsa-miR-223-5p	AIMP1
hsa-miR-223-5p	TRAF5
hsa-miR-223-5p	EGF
hsa-miR-23a-3p	TJP1
hsa-miR-23a-3p	HMGB1
hsa-miR-23a-3p	AHR
hsa-miR-23a-3p	TGFBR3
hsa-miR-23a-3p	ANXA11
hsa-miR-23a-3p	NR3C1
hsa-miR-23a-3p	HLA-A
hsa-miR-23a-3p	IFNG
hsa-miR-23a-3p	IL6ST
hsa-miR-23a-3p	CD46
hsa-miR-23a-3p	MYD88

hsa-miR-23a-3p	NFE2L2
hsa-miR-23a-3p	PIK3CB
hsa-miR-23a-3p	MAPK1
hsa-miR-23a-3p	VIP
hsa-miR-23a-3p	RNF19A
hsa-miR-23a-3p	KIAA1109
hsa-miR-23a-3p	ATN1
hsa-miR-23a-3p	TRAF5
hsa-miR-23a-3p	VCAN
hsa-miR-23a-3p	AQP4
hsa-miR-23a-3p	HLA-C
hsa-miR-23a-3p	IFNB1
hsa-miR-23a-3p	HLA-B
hsa-miR-23a-3p	ARIH1
hsa-miR-23a-3p	ATF7IP
hsa-miR-23a-3p	DNER
hsa-miR-223-3p	VEGFA
hsa-miR-223-3p	RASGRP1
hsa-miR-223-3p	PIK3CD
hsa-miR-155-5p	MTX1
hsa-miR-155-5p	VEGFA
hsa-miR-155-5p	FAS
hsa-miR-155-5p	SOCS5
hsa-miR-155-5p	TLR4
hsa-miR-155-5p	TGFB2
hsa-miR-155-5p	RELA
hsa-miR-155-5p	ANXA1
hsa-miR-155-5p	CD274
hsa-miR-155-5p	RASGRP1
hsa-miR-155-5p	CXCR4
hsa-miR-155-5p	EGR2
hsa-miR-155-5p	KLF4
hsa-miR-155-5p	CFH
hsa-miR-155-5p	SOD2
hsa-miR-155-5p	ANXA11
hsa-miR-155-5p	ADM
hsa-miR-155-5p	AMD1
hsa-miR-155-5p	XIAP
hsa-miR-155-5p	CRK
hsa-miR-155-5p	CYP24A1
hsa-miR-155-5p	GEM
hsa-miR-155-5p	HMOX1
hsa-miR-155-5p	IL1B
hsa-miR-155-5p	IL1RAP
hsa-miR-155-5p	IL6ST
hsa-miR-155-5p	IL10

hsa-miR-155-5p	IL15
hsa-miR-155-5p	IL18
hsa-miR-155-5p	CD46
hsa-miR-155-5p	NR3C2
hsa-miR-155-5p	NFE2L2
hsa-miR-155-5p	PIK3CB
hsa-miR-155-5p	PLXNA2
hsa-miR-155-5p	MAPK1
hsa-miR-155-5p	PTPRF
hsa-miR-155-5p	TNF
hsa-miR-155-5p	VIP
hsa-miR-155-5p	AHSA1
hsa-miR-155-5p	SMUG1
hsa-miR-155-5p	RNF19A
hsa-miR-155-5p	ALB
hsa-miR-155-5p	ATN1
hsa-miR-155-5p	VCAN
hsa-miR-155-5p	ANGPT1
hsa-miR-155-5p	EGF
hsa-miR-155-5p	AQP1
hsa-miR-155-5p	ESD
hsa-miR-155-5p	HLA-C
hsa-miR-155-5p	EPCAM
hsa-miR-155-5p	BRAF
hsa-miR-155-5p	HLA-B
hsa-miR-155-5p	LCN2
hsa-miR-155-5p	ARIH1
hsa-miR-155-5p	PRKCD
hsa-miR-155-5p	PLA2G15
hsa-miR-155-5p	MBTPS2
hsa-miR-155-5p	ATF7IP
hsa-miR-155-5p	RTN4R
hsa-miR-155-5p	IL17D
hsa-miR-155-5p	IL23R
hsa-miR-125a-5p	P2RX7
hsa-miR-125a-5p	SOD2
hsa-miR-125a-5p	AMD1
hsa-miR-125a-5p	XIAP
hsa-miR-125a-5p	IL6ST
hsa-miR-125a-5p	NFE2L2
hsa-miR-125a-5p	SCAF11
hsa-miR-125a-5p	KIAA1109
hsa-miR-125a-5p	VCAN
hsa-miR-125a-5p	PTPN2
hsa-miR-125a-5p	MAP6
hsa-miR-140-5p	ETS1

hsa-miR-140-5p	TCF4
hsa-miR-140-5p	RNF19A
hsa-miR-140-5p	POLDIP2
hsa-miR-140-5p	DPYSL5
hsa-miR-140-5p	EPRS1
hsa-miR-29a-3p	TJP1
hsa-miR-29a-3p	TGFB2
hsa-miR-29a-3p	RELA
hsa-miR-29a-3p	SIRT1
hsa-miR-29a-3p	AKR1B1
hsa-miR-29a-3p	EXTL3
hsa-miR-29a-3p	NR3C1
hsa-miR-29a-3p	CD46
hsa-miR-29a-3p	NR3C2
hsa-miR-29a-3p	NFE2L2
hsa-miR-29a-3p	PTPRF
hsa-miR-29a-3p	TCF4
hsa-miR-29a-3p	AIMP2
hsa-miR-29a-3p	SCAF11
hsa-miR-29a-3p	RNF19A
hsa-miR-29a-3p	POLDIP2
hsa-miR-29a-3p	TRIB2
hsa-miR-29a-3p	ERAP1
hsa-miR-29a-3p	DPYSL5
hsa-miR-29a-3p	ADO
hsa-miR-29a-3p	TRAF5
hsa-miR-29a-3p	TNFRSF1A
hsa-miR-29a-3p	VCAN
hsa-miR-29a-3p	AQP1
hsa-miR-29a-3p	CKB
hsa-miR-29a-3p	CXCL8
hsa-miR-29a-3p	EPRS1
hsa-miR-146a-5p	VEGFA
hsa-miR-146a-5p	ICOS
hsa-miR-146a-5p	SOCS5
hsa-miR-146a-5p	SOCS3
hsa-miR-146a-5p	TGFB2
hsa-miR-146a-5p	SOCS1
hsa-miR-146a-5p	ASS1
hsa-miR-146a-5p	CD274
hsa-miR-146a-5p	RASGRP1
hsa-miR-146a-5p	KLF4
hsa-miR-146a-5p	SOD2
hsa-miR-146a-5p	ANXA11
hsa-miR-146a-5p	CRYAA
hsa-miR-146a-5p	CYP27B1
hsa-miR-146a-5p	CFI
hsa-miR-146a-5p	IL18
hsa-miR-146a-5p	NOS2
hsa-miR-146a-5p	PIK3CA
hsa-miR-146a-5p	PIK3CG
hsa-miR-146a-5p	MAPK3
hsa-miR-146a-5p	PTPRF
hsa-miR-146a-5p	SAG
hsa-miR-146a-5p	CCL20
hsa-miR-146a-5p	TM7SF2
hsa-miR-146a-5p	SMUG1
hsa-miR-146a-5p	ERAP1
hsa-miR-146a-5p	CXCL16
hsa-miR-146a-5p	NLRC4
hsa-miR-146a-5p	ERAP2
hsa-miR-146a-5p	CXCL10
hsa-miR-146a-5p	PSMB9
hsa-miR-146a-5p	TNFRSF1A
hsa-miR-146a-5p	VCAN
hsa-miR-146a-5p	ACAN
hsa-miR-146a-5p	GPR35
hsa-miR-146a-5p	PTPN2
hsa-miR-146a-5p	CD40
hsa-miR-146a-5p	AIF1
hsa-miR-146a-5p	CKB
hsa-miR-146a-5p	HLA-C
hsa-miR-146a-5p	IFNB1
hsa-miR-146a-5p	BRAF
hsa-miR-146a-5p	PDCD1
hsa-miR-146a-5p	HLA-B
hsa-miR-146a-5p	LCN2
hsa-miR-146a-5p	ARIH1
hsa-miR-146a-5p	RTN4R
hsa-miR-146a-5p	MAP2K7
hsa-miR-146a-5p	MZB1
hsa-miR-146a-5p	IFNL2
hsa-miR-491-5p	ATN1
hsa-miR-193a-5p	ANXA11
hsa-miR-193a-5p	ERAP1
hsa-miR-193a-5p	ACAN
hsa-miR-193a-5p	EPRS1
hsa-miR-223-5p	ETS1
hsa-miR-223-5p	CRK
hsa-miR-223-5p	ERAP2

Genes (142)	AIMP1	HLA-A	IL15	TRIB2
ETS1	SLC9A3R2	CD46	IL18	ERAP1
MyD88	EPRS1	NFE2L2	NR3C2	ADO
SOCS1	ZEB2	PIK3CB	PLXNA2	TNFRSF1A
VEGFA	IL1RN	MAPK1	PTPRF	CKB
PTEN	CRK	VIP	TNF	ICOS
CXCR4	IFNG	RNF19A	AHSA1	ASS1
CFH	PIK3CG	KIAA1109	SMUG1	CRYAA
STAT1	CCL5	ATN1	ALB	CYP27B1
FAS	MAP2K7	VCAN	ANGPT1	CFI
SOD2	ARIH1	AQP4	AQP1	NOS2
XIAP	AHR	IFNB1	EPCAM	MAPK3
CCL8	RASGRP1	HLA-B	BRAF	SAG
CXCL8	KLF4	ATF7IP	LCN2	CCL20
IL6	S100B	DNER	PRKCD	TM7SF2
IL6ST	TLR4	PIK3CD	PLA2G15	CXCL16
NLRP3	TLR2	MTX1	MBTPS2	NLRC4
TRPV2	IL1RAP	SOCS5	RTN4R	ERAP2
MAPK14	DECR1	TGFB2	IL17D	CXCL10
TJP1	ESD	RELA	IL23R	PSMB9
SOCS3	GRAP2	ANXA1	P2RX7	ACAN
SIRT1	S100A12	CD274	SCAF11	GPR35
ICAM1	HLA-C	EGR2	PTPN2	CD40
CTLA4	AMD1	ADM	MAP6	AIF1
NR3C1	TRAF5	CYP24A1	POLDIP2	PDCD1
IL2	EGF	GEM	DPYSL5	MZB1
PIK3CA	HMGB1	HMOX1	AKR1B1	IFNL2
CCL2	TGFBR3	IL1B	EXTL3	
TCF4	ANXA11	IL10	AIMP2	
